# Controlled co-delivery of anti-inflammatory drugs from bilayer polymer films coating a meniscus implant

**DOI:** 10.1007/s13346-025-01942-5

**Published:** 2025-08-25

**Authors:** Alfonso F. Blanco, Gustavo Lou, Alba Pensado-López, Aldo Ummarino, Fernando Torres Andón, José Crecente-Campo, María José Alonso

**Affiliations:** 1 Center for Research in Molecular Medicine and Chronic Diseases (CiMUS), University of Santiago de Compostela, Health Research Institute of Santiago de Compostela (IDIS), Santiago de Compostela, Spain; 2 Department of Pharmacy and Pharmaceutical Technology, School of Pharmacy, University of Santiago de Compostela, Santiago de Compostela, Spain; 3 Department of Biomedical Sciences, Humanitas University, Pieve Emanuele, Milan, 20072 Italy; 4 IRCCS Humanitas Research Hospital, Rozzano, Milan, 20089 Italy; 5 Medical Oncology Unit, Instituto de Investigación Biomédica de A Coruña (INIBIC), Complexo Hospitalario de A Coruña (CHUAC), Coruña, 15006 A Spain; 6 Instituto Madrileño de Estudios Avanzados en Nanociencia (IMDEA Nanociencia), Madrid, Spain

**Keywords:** Knee osteoarthritis, Meniscus implant, Controlled drug release, Polymer, Anti-inflammatory drug, Inflammation

## Abstract

Knee osteoarthritis (OA), a degenerative joint disease, is increasingly prevalent worldwide and often results from a meniscal deterioration that leads to meniscus removal. Replacing the damaged meniscus with a non-biodegradable prosthesis offers an innovative solution to prevent OA progression, particularly in older patients. However, the long-term use of anti-inflammatory drugs for pain relief and prosthesis integration can cause severe off-target side effects. The objective of this work was to design and develop drug-loaded bilayer polymer films to be used as coatings for a meniscus polycarbonate urethane (PCU). The developed bilayer polymer films enabled a sustained release of two anti-inflammatory drugs - dexamethasone (DEX) and celecoxib (CLX) - with distinct release kinetics (1–4 weeks for DEX and 6–9 months for CLX). This release profile was defined to modulate post-surgical and chronic inflammation within the knee joint, respectively. Two bilayer prototypes showed consistent biodegradation, drug release, drug loading, and reproducibility. Furthermore, the systems were sterile, biocompatible, and maintained the anti-inflammatory efficacy of the released drugs, effectively reducing pro-inflammatory cytokine secretion from human primary macrophages.

**Supplementary Information:**

The online version contains supplementary material available at 10.1007/s13346-025-01942-5.

## Graphical abstract

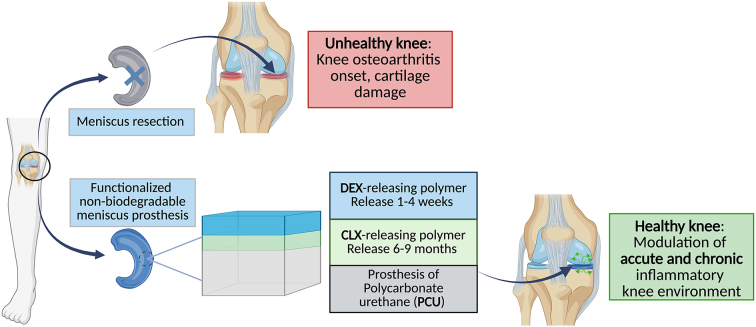

## Introduction

Osteoarthritis (OA) is a multifactorial, degenerative, and chronic joint disease characterized by progressive cartilage degradation, bone remodeling, and an imbalance between synthesis and degradation processes, leading to pain, inflammation, stiffness, and loss of function [1]. It is the most common form of arthritis and a major cause of disability, affecting over 650 million individuals worldwide in 2020 [2]. Knee OA is particularly prevalent, with its increasing incidence attributed to longer life expectancy and rising obesity rates, the latter being a significant risk factor [3].

The meniscus plays a crucial role in knee function by providing stabilization, load distribution, and shock absorption [4–6]. However, meniscal injuries are common and often fail to heal due to the avascular nature of the inner meniscus, necessitating surgical intervention [7, 8]. While partial meniscectomy is frequently performed to alleviate pain, it tends to accelerate joint degeneration and significantly increases the risk of OA development [9]. The use of meniscus implants or scaffolds that promote regeneration is a promising alternative to avoid the onset of OA after a meniscectomy [10]. To address this, meniscus implants and scaffolds have been developed to restore joint biomechanics and delay disease progression. Among them, the non-biodegradable NUsurface^®^ prosthesis (Active Implants, Israel) serves as an artificial meniscus substitute after total meniscectomy. Approved in several European countries and Israel, it is currently undergoing FDA clinical trials [11, 12]. Despite its advantages, implantation of the NUsurface^®^ prosthesis could induce a foreign body reaction (FBR), requiring anti-inflammatory treatment to mitigate the patient’s pain, post-surgical inflammation and improve implant integration [13, 14]. Current anti-inflammatory drugs have important limitations; for example systemic administration of COX-2 inhibitors, such as celecoxib (CLX), can cause gastrointestinal, renal, and cardiovascular side effects [15, 16], whereas intra-articular (IA) corticosteroid injections, such as dexamethasone (DEX), provide short-term relief but pose risks of infection and patient discomfort [17]. To overcome these challenges, localized and sustained drug delivery strategies are needed to enhance therapeutic efficacy while minimizing systemic and local adverse effects [18].

Polymer coatings have been widely explored to improve implant biocompatibility, facilitate tissue integration, and enable controlled drug release. An optimized coating can provide site-specific drug release, shielding drugs from enzymatic degradation while reducing systemic toxicity [19, 20]. CLX, a potential disease-modifying osteoarthritis drug (DMOAD), has shown protective effects in OA models by reducing inflammation and cartilage degradation [21–25], whereas a low-dose IA administration of DEX has shown chondroprotective potential when delivered IA, and its potential consideration as a DMOAD has been suggested. Therefore, the hypothesis of this study has been that a polymer bilayer coating enabling the sustained release of two drugs widely used in OA treatment, CLX for long-term inflammation control and short-term release of DEX for early-phase inflammation management, may improve implant performance and clinical outcomes [24, 26]. Within this context, the current challenge in the field of meniscus-related degenerative pathologies is to design in situ therapies enabling controlled drug release with the goal of achieving a prolonged response and a reduction of systemic side effects [27].

In this study, we developed a bilayer polymer coating for the NUsurface^®^ prosthesis intended to release these two anti-inflammatory drugs with distinct, controlled release kinetics. Using square-shaped polycarbonate urethane (PCU) implants (0.7 × 0.7 × 0.3 cm), we screened various polymer formulations to optimize controlled DEX release for short-term inflammation control (1–4 weeks) and CLX release for long-term pain and inflammation management (6–9 months). The coatings were characterized for their drug release kinetics, degradation profiles and biocompatibility. To assess their anti-inflammatory efficacy, we conducted in vitro studies in primary human monocyte-derived macrophages (HMDMs). Our findings provide a foundation for the development of an implantable, bioactive, drug-releasing meniscus prosthesis designed to enhance post-surgical outcomes and reduce OA progression.

## Materials and methods

### Materials

Poly(DL-lactide-co-glycolide) (PLGA), of two different lactic acid: glycolic acid (LA: GA) ratios (PLGA 50:50, and PLGA 85:15), poly(caprolactone) (PCL) of low (LMW-PCL) and high molecular weight (HMW-PCL), poly(L-lactide) (PLLA) of low (LMW-PLLA) and high molecular weight (HMW-PLLA), and poly(lactic acid)-poly(ethylene glycol) di-block copolymer (PLA-PEG) were purchased from Evonik Industries (Darmstadt, Germany). PLGA and PLLA were ester-terminated. The MW of PEG was 5,000 Da (5,000 g/mol). PLA-PEG copolymers are typically synthesized by ring-opening polymerization of lactide using PEG as the initiator, as reported in the literature [28, 29]. LMW-PLGA LA: GA ratio 50:50 was obtained from PolySciTech, a division of Akina (Indiana, USA). Sodium azide (NaN_3_) was purchased from Sigma-Aldrich (Missouri, USA). Acetone was obtained from Fisher chemicals (New Hampshire, USA). Dichloromethane (DCM and acetonitrile (ACN) were distributed by Scharlau (Barcelona, Spain). Phosphate saline buffer (PBS), Tween^®^80, trifluoracetic acid (TFA) and methanol (MeOH) were supplied by Scientific (Nottingham, England), Merck (Darmstadt, Germany), Sigma-Aldrich (Missouri, USA) and VWR Chemicals (Pennsylvania, USA), respectively. The square-shaped implants made of polycarbonate urethane (PCU) were kindly donated by Active Implants (Israel).

### Drug solubility in release buffer

An excess of the drugs (DEX and CLX) was incubated in agitation (700 rpm) in 1 mL of PBS supplemented with Tween^®^80 concentrations ranging from 0 to 1% (w/v) for 24 h. Then, samples were centrifuged at 10,000 rpm for 20 min, the supernatant was diluted in MeOH: H_2_O 65:35 (v/v), and the solutions were quantified by ultraperformance liquid chromatography (UPLC) with a TUV detector at 239 nm with a column Kinetex^®^ 1.7 μm C18 100 Å, LC Column 50 × 2.1 mm acquired from Phenomenex (Torrance, CA, USA), maintaining the samples at 20 °C in a Waters Acquity H-Class UPLC system (Waters, Milford, USA).

### Production of drug-releasing polymer films

To prepare the polymer-drug solutions, appropriate amounts of each drug (DEX or CLX) were dissolved in acetone and DCM, respectively [30–32]. In DEX-releasing polymers, PLGA (PLGA 50:50; intrinsic viscosity (IV) = 0.32–0.44 dL/g and PLGA 85:15; IV = 1.3–1.7 dL/g), LMW-PLGA (PLGA 50:50; MW = 8 kDa), and PLA-PEG (MW = 5.3 kDa) were dissolved to their final concentrations using acetone solutions of DEX of determined concentrations. Regarding CLX-loaded polymers, the polymers PCL, with intrinsic viscosities of 0.39 dL/g (~ 32 KDa; “LMW-PCL”) and 0.9 dL/g (~ 73 KDa; “HMW-PCL”) and PLLA, with IV of 1.0 dL/g (~ 74 KDa; “LMW-PLLA”) and 2.9 dL/g (~ 210 KDa; “HMW-PLLA”) as well as combinations of LMW-PCL (IV = 0.39 dL/g ~ 32 kDa) and HMW-PLLA (IV = 2.9 dL/g ~ 210 kDa) were dissolved to their final concentrations using DCM solutions of CLX of determined concentrations. The polymers used were selected based on their hydrophobicity and degradation rates. All these procedures were carried out under ambient conditions. These films were prepared by solvent casting. 70 µL of polymer-drug solutions were then cast on square-shaped meniscus implants (made of PCU) of 0.7 × 0.7 × 0.3 cm. The organic solvent was allowed to evaporate for 1 h at room temperature. The resulting drug-loaded polymer films were then vacuum-dried for at least 24 h (Supplementary Fig. 1A).

### Production of bilayer drug-releasing polymer coatings

To prepare the polymer-drug solutions, appropriate amounts of each drug (DEX or CLX) to achieve the desired concentrations were dissolved in acetone and DCM, respectively [30–32]. Polymers, LMW-PLGA (PLGA 50:50; MW = 6.9 kDa), and PLA-PEG (MW = 5.3 kDa) were dissolved at a concentration of 200 mg/mL using a solution of DEX of 5 mg/mL. Regarding CLX-releasing polymers, blends of PLLA (IV = 2.9 dL/g; MW ~ 210 kDa) and PCL (IV = 0.39 dL/g; MW ~ 32 kDa) were dissolved to their final concentrations using a solution of CLX of 30 mg/mL. The square-shaped PCU implants (0.7 × 0.7 × 0.3 cm), held in place by a needle, were immersed in the polymer-CLX solution and immediately withdrawn, allowing 10 min for drying. The cycle was repeated a total of 3 times. After an additional 3 h of drying, 5 new immersion cycles were performed, in this case in the polymer-DEX solution and with a drying time of 15 min between cycles. Finally, the organic solvent was allowed to evaporate for 72 h (Supplementary Fig. 1C). Manual rotation between dips was performed. Although slight differences in polymer accumulation may occur on the larger faces or the face in contact with the needle during dipping, the visual appearance of the coating was homogeneous across all surfaces.

### Drug release evaluation

Drug release studies were performed in agitation (450 rpm) at 37 °C in PBS Tween^®^80 1% (w/v) to ensure sink conditions. The amount of drug (DEX and CLX) released was quantified by reverse phase ultra-performance liquid chromatography (UPLC) with a TUV detector at 239 nm using a column Kinetex^®^ 1.7 μm C18 100 Å, LC Column 50 × 2.1 mm acquired from Phenomenex (Torrance, CA, USA), maintaining the samples at 20 °C in a Waters Acquity H-Class UPLC system (Waters, Milford, USA) [33]. The mobile phase consisted of A: deionized H_2_O acidified with TFA 0.1% (v/v) and B: ACN acidified with TFA 0.1% (v/v) pumped with a flow rate of 0.1 mL/min. The injection volume was 5 µL, and the column oven temperature was set to 40 °C. To control the UPLC/UV system as well as for data acquisition and processing, EMPOWER software was used. To quantify the amount of DEX and CLX, a calibration curve, ranging from 1 to 100 ppm was performed. The curves had a correlation coefficient (R^2^) of 1 for DEX, and 0.9999 for CLX (*n* = 22). The validation procedure was carried out according to the ICH guidelines [33, 34]. Limit of detection (LOD) and quantification (LOQ) were calculated directly from the calibration plots, as 3.3σ/S and 10σ/S, respectively, where σ is the standard deviation of intercept and S is the slope of the calibration plot [34]. The values were LOD = 1.73 ppm, and LOQ = 5.25 ppm for DEX, and LOD = 0.3 ppm, and LOQ = 0.92 ppm for CLX. All analyzed samples were clear and free of visible particles. Chromatographic peaks matched pure drug standards, and no signal interference or column pressure issues were observed throughout the study.

### Drug loading assay

PCU prostheses coated with drug-releasing polymers were immersed in 5 mL of a mixture of DCM/acetone 3/2 (v/v) for up to 24 h to ensure polymer disolution. Samples of 200 µL were then transferred to microtubes containing 800 µL methanol and centrifuged at 10,000 rpm for 20 min. 200 µL of supernatant were further diluted with 800 µL MeOH: H_2_O 65:35 (v/v) and the concentration of drug was quantified by UPLC with a TUV detector at 239 nm using a column Kinetex^®^ 1.7 μm C18 100 Å, LC Column 50 × 2.1 mm acquired from Phenomenex (Torrance, CA, USA), maintaining the samples at 20 °C in a Waters Acquity H-Class UPLC system (Waters, Milford, USA) [33]. The mobile phase consisted of A: deionized H_2_O acidified with TFA 0.1% (v/v) and B: ACN acidified with TFA 0.1% (v/v) pumped with a flow rate of 0.1 mL/min. The gradient was from 25 to 60% of B in 6.5 min, and 6 min from 60 to 25% of B. The injection volume was 5 µL, and the column oven temperature was set to 40 °C. To control the UPLC/UV system as well as for data acquisition and processing, EMPOWER software was used. To quantify the amount of DEX and CLX, a calibration curve, ranging from 1 to 100 ppm was used. The release buffer had no matrix effect on the quantification of the drugs. The curves had a correlation coefficient (R^2^) of 1 for DEX, and 1 for CLX (*n* = 8). The values were LOD = 0.383 ppm, and LOQ = 1.162 ppm for DEX, and LOD = 0.187 ppm, and LOQ = 0.567 ppm for CLX. All analyzed samples were clear and free of visible particles. Chromatographic peaks matched pure drug standards, and no signal interference or column pressure issues were observed throughout the study.

### Characterization of the drug-releasing polymer films

#### Differential scanning calorimetry (DSC)

DSC measurements for polymers, drugs, and combinations of polymer and drug were recorded with a DSC Q1000 V9.9 (TA Instruments, New Castle, DE, USA) in standard aluminum sample pans. The samples were heated from 20 °C to 400 °C, depending on the sample analyzed, with a heating rate of 10 °C/min in a nitrogen atmosphere. Data recording and processing of the first heating cycles were carried out with the software Advantage (TA Instruments, New Castle, DE, USA).

#### Powder X-ray diffraction (XRD)

Diffraction measurements of crystalline powder were carried out by an Empyrean diffractometer of the PANAlytical brand. The X-rays were obtained from a sealed tube with Cu anode (λ(Kα1) = 1.5406 Å) and were collimated prior to incidence on the sample with optics including a W/Si bilayer mirror. The radiation emitted by the sample was collected with a “PIXcel3D” type solid state detector. The samples were mounted at ambient temperature on a flat base without signal (Si single crystal), to avoid the amorphous component different from that coming from the sample. Diffractograms were taken in an angular range of 2 to 40° with a step of 0.04 and a time per step of 8 s. To perform the mathematical adjustments of the obtained diffractograms, the program HighScore Plus: Version 3.0d was used.

### Degradation of bilayer drug-releasing polymer coatings

Drug-releasing polymer coated PCU implants were immersed in a volume of 10 mL of PBS (pH 7.4) supplemented with 1% (w/v) Tween^®^80. Buffer was not refreshed during the study to allow monitoring of natural pH changes due to polymer degradation. The 10 mL PBS volume provided sufficient buffering capacity relative to the sample size (0.7 × 0.7 × 0.3 cm). At defined time points, the coated squared-shaped PCU implants were removed from the release buffer for further analysis:

#### Field-emission scanning electron microscopy (FESEM)

Drug-releasing polymer coatings were sputter coated with a layer of iridium and imaged in a Zeiss UltraPlus analytical FESEM with a beam voltage of 3 kV and a magnification ranging from 500 to 10,000X for the analysis of the surface of the coatings. Also, Zeiss EVO analytical FESEM with a beam voltage of 20 kV and magnification ranging from 60X to 5,000X was used for the measure of the coating thickness and analyzing side profile upon degradation after sagittal cut.

#### pH measurements

pH was measured in the media where the polymer coated PCU implants were incubated over specific periods using a pH meter calibrated using standard buffer solutions of known pH values ranging from pH 2.0 to 10.0.

### In vitro studies

#### Drug release evaluation

Drug release studies to evaluate released drug activity in vitro were performed at 37 °C in PBS Tween^®^80 0.05% (w/v) supplemented with penicillin/streptomycin 1% (v/v). The amount of drug (DEX and CLX) released was quantified by UPLC using the same method as described in section Drug loading assay. The release buffer collected and quantified at each time point was subsequently lyophilized. Afterwards, the powder was resuspended in sterilized miliQ water and evaluated in vitro.

Stored release buffers were diluted to achieve a concentration of DEX and CLX (Table 1) as close as possible to their active anti-inflammatory concentration (10 µM for DEX and 25 µM for CLX), based on previous investigations [26]. A minimum dilution of 1:10 using RPMI was implemented for each sample, regardless of the concentration of drugs quantified in the media (Supplementary Fig. 2), to prevent possible toxicity by the release buffer (Tween80 0.05% w/v).

**Table 1:** Concentrations of DEX and CLX obtained from prototype PLA-PEG and prototype PLGA bilayer polymer coatings and total percentage (%) of drug released at each time point for the in vitro validation of their sterility and anti-inflammatory activity, and efficacy

		CLX			DEX			
Prototype	Time point	ppm	µM	% released	ppm	µM	% released	Dilution
Prototype PLA-PEG	3 h	27.27	71.50	0.71 ± 0.22	193.63	493.38	39.08 ± 1.01	1:50
	3 d	118.75	311.38	5.58 ± 0.97	97.58	248.65	89.16 ± 3.67	1:20
	1 w	58.13	152.44	7.38 ± 1.01	22.95	58.49	93.70 ± 3.06	1:20
	2 w	130.69	342.70	11.20 ± 1.16	17.41	44.36	96.63 ± 1.40	1:10
	4 w	134.31	352.17	20.37 ± 1.58	1.81	4.60	~ 100	1:15
Prototype PLGA	3 h	7.14	18.74	0.21 ± 0.02	16.40	41.80	6.28 ± 0.31	1:10
	3 d	40.81	107.00	2.81 ± 0.32	45.82	116.74	44.76 ± 1.76	1:10
	1 w	60.53	158.73	4.87 ± 0.31	48.45	123.46	63.51 ± 1.03	1:10
	2 w	108.59	284.74	8.49 ± 1.87	25.69	63.46	72.61 ± 2.87	1:10
	4 w	115.87	303.84	16.08 ± 3.44	10.49	26.75	82.77 ± 5.89	1:12

**Abbreviations**: CLX: Celecoxib. DEX: Dexamethasone. PLA-PEG: poly(lactic acid)-poly(ethylene glycol) di-block copolymer. PLGA: Poly(lactic-co-glycolic) acid. ppm: parts per million (µg/mL). h: hour. d: days. w: week. µM: Micromolar. Values represent the mean ± standard deviation (*n* = 3)

#### In vitro control of endotoxin contamination

The chromogenic LAL-test (LO50650U, Lonza) was used, following the manufacturer’s indications. Each of the samples used for in vitro studies showed endotoxin levels below 0,125 EU/ml (otherwise the samples were discarded) to prevent possible interferences in their immunotoxicity activity.

#### Monocyte isolation, differentiation, and treatment of human primary macrophages

Monocytes were isolated from the whole blood of six healthy donors (buffy coats) following the Ficoll and Percoll separation method, as previously described [35], and subsequently counted with Trypan Blue staining 0.04% using a counting chamber (Kova international, BVS100H). In order to obtain human monocyte-derived macrophages (HMDMs), monocytes were resuspended in RPMI supplemented with penicillin/streptomycin and glutamine, subsequently counted, and 10^6^ cells were plated in each well of 24-well plates to allow their attachment to the plate. After 30 min of incubation at 37 °C, media was removed, and a new media containing human recombinant M-colony stimulating factor (hrM-CSF, Peptrotech, 300 − 25) supplemented with fetal bovine serum (FBS), penicillin/streptomycin and glutamine were added for differentiation towards macrophages after 5 days.

At day 5, macrophages were treated with appropriately diluted release buffers. Samples were added together with complete media for 24 h. Non-treated HMDMs were used as the negative control, while HMDMs treated with LPS + IFNγ (100 ng/mL and 50 ng/mL, respectively) were used as the positive control (M1-like macrophages). All other samples were treated with free drugs (DEX, CLX, and DEX + CLX) at appropriate concentrations (10 µM for DEX and 25 µM for CLX) for comparison with equivalent concentrations of the drugs in release buffer from Prototype PLA-PEG or Prototype PLGA drug-releasing bilayer polymer coatings at determined times.

#### Evaluation of biocompatibility and toxicity

To assess the biocompatibility of the drug-loaded polymer coatings using primary human macrophages, the AlamarBlue™ cell viability assay was performed. After exposure of macrophages to conditions described in Table 1 for 24 h (relative day 6 of cell culture), which corresponds to the release buffer containing the drugs and products of polymer degradation at different time points, supernatants were collected for cytokine quantification, while 1 mL of AlamarBlue™ HS Cell Viability Reagent 10% (Invitrogen, A50101) was added to the remaining cells in each well of the 24-well plates, following the manufacturer’s protocol. Plates were incubated for 3 h at 37 °C, protected from light. 100 µL from each well, in triplicate, were transferred to black 96-well plates, and fluorescence was measured at 560–590 nm using a Synergy H4 Microplate reader (BioTek).

Non-treated cells were used as controls and considered as 100% cell viability. Cell viability was calculated according to the equation: % Cell viability = (Sample Fluorescence / Control Fluorescence) × 100.

#### Assessment of anti-inflammatory activity and efficacy by measurement of cytokine secretion

After checking the absence of LPS contamination and non-toxic activity of the release buffer containing DEX and CLX at indicated time points, the anti-inflammatory activity of the drugs in the release buffer was compared with the free drugs at equivalent concentrations by ELISA. The anti-inflammatory activity was evaluated by quantifying the secretion of relevant inflammatory signals (TNF-α, CCL2, and PGE2) from primary human macrophages treated with stimulated with LPS + IFNγ alone, in combination with free drugs or exposed to the release buffer for 24 h.

ELISAs were performed using the commercial kit DuoSet^®^ ELISA Development Systems (Bio-Techne) for TNF-α (DY210) and the Prostaglandin E2 ELISA kit (Cayman) for PGE2 (004CA514010-96), following the manufacturer’s instructions. ELISA assays for CCL2 were performed using a custom kit developed in the HUNIMED lab [35].

The amount of cytokine secretion (ng/ml) in macrophages treated with LPS + IFNγ (positive control) was normalized to 100% and, subsequently, values for the remaining samples were normalized and expressed as percentage (%) of control, according to the equation: % of Control = (ng/ml of Sample / ng/ml of Control) × 100.

## Results and discussion

Our aim was to engineer a biodegradable bilayer polymer coating around a polycarbonate urethane (PCU) meniscus prosthesis to release two anti-inflammatory drugs with controlled release kinetics. More precisely, dexamethasone (DEX) was intended to be released within 1–4 weeks to alleviate the acute inflammation caused mainly by the surgery. Meanwhile, celecoxib (CLX) was intended to be released within 6–9 months to relieve the chronic inflammation related to the prosthesis. Both drugs are expected to mediate the incorporation of the implant into the knee cavity, reducing potential adverse inflammatory reactions. Therefore, we developed and optimized the preparation of the bilayer coating, and we performed experiments to evaluate the activity of released drugs in vitro. For this, we followed a specific work plan consisting of:

1. The screening of different biodegradable and biocompatible polymers for the independent release of DEX and CLX with the desired release kinetics.
2. The development of drug-releasing bilayer polymer coatings results from the combination of the most promising prototypes selected for the independent release of the drugs. This development consisted of evaluating the capacity of the bilayer systems to release CLX and DEX in a required time frame, characterization of their physicochemical properties, and in vitro study of their degradation.
3. The in vitro evaluation of the sterility and biocompatibility of the drugs and polymer degradation products from the bilayer coated system, and the anti-inflammatory activity of these drugs in human primary macrophages.

### Design and optimization of drug-loaded polymer films

Polyesters were selected as the base polymers of the bilayer coating due to their controlled biodegradability, tunable release properties and favorable regulatory profile. The primary objective of this section was to evaluate the influence of the polymer type on the drug release profile while keeping other parameters—such as the preparation method and the number of polymer layers—constant. Although the ultimate goal was to achieve the controlled release of two different anti-inflammatory drugs from the same meniscus prosthesis with distinct release kinetics, the initial screening focused on evaluating the release of each drug independently within a single polymeric matrix.

#### CLX-loaded polymer films

**Fig. 1:**
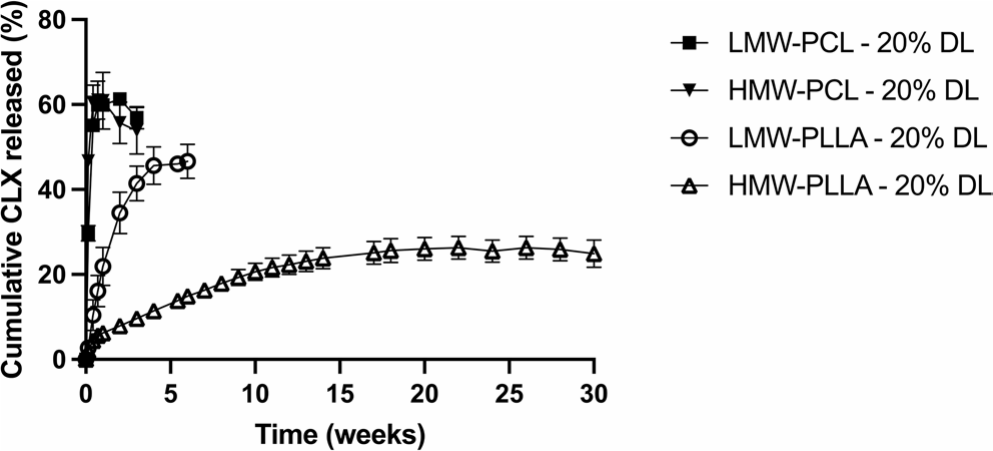
Release kinetics of CLX expressed as the total percentage of drug released (%) from polymer films composed of polymers PCL and PLLA, both HMW and LMW, at a concentration of 50 mg/mL, with CLX loadings of 20%. **Abbreviations**: CLX: Celecoxib. LMW: Low molecular weight. HMW: High molecular weight. DL: Drug loading. PLLA: poly(L-lactide) (PLLA). PCL: poly(caprolactone). Values represent the mean ± standard deviation (*n* = 3). Note: In cases where drug release was minimal between sampling points (e.g., HMW-PCL and LMW-PCL), slight decreases in cumulative release were observed due to sample replacement with fresh buffer, which may dilute the drug concentration when no measurable release occurred. This is a known limitation of partial-volume sampling methods

Poly(caprolactone) (PCL) and poly(L-lactide) (PLLA) were chosen due to their slow degradation rate and, hence, long-term release capacity of CLX (6 to 9 months). This profile was expected to modulate a process of chronic inflammation [36, 37]. The influence of molecular weight (MW) on degradation rate was explored by testing high molecular weight (HMW) (~ 210 kDa HMW-PLLA and ~ 73 kDa HMW-PCL) and low molecular weight (LMW) (~ 74 kDa LMW-PLLA and ~ 32 kDa LMW-PCL) variants of each polymer [38–42]. CLX-loaded films were fabricated by dissolving CLX and each polymer in dichloromethane (DCM) and casting the solution onto square-shaped PCU implants. After curing, the films and PCU implants were immersed in PBS with 1% (w/v) Tween^®^80, and CLX release was monitored via UPLC (Fig. 1). The drug release study was halted when a plateau release phase was observed.

The release studies revealed that CLX was consistently released faster from PCL films, irrespective of MW, as compared to from PLLA films. PLLA exhibited MW-dependent release kinetics, with HMW-PLLA providing more sustained release compared to LMW-PLLA [40]. The differences in release kinetics can be explained by the polymer chain length. Since none of these polymers is expected to degrade within a few weeks, the observed release behavior aligns with previous studies linking polymer chain length to drug diffusion [43]. Moreover, this faster release may also be explained by increased polymer swelling and water uptake in LMW systems, which create transient pathways for drug diffusion due to reduced chain entanglement and matrix stiffness [44]. The denser matrix created by HMW-PLLA restricted water penetration, slowing degradation and drug diffusion and, therefore, delaying drug release. As expected, due to the larger difference in MW between variants, these effects are more pronounced in PLLA films (~ 140 kDa) than in PCL films (~ 41 kDa). In addition to polymer chain length, the degree of crystallinity also affects water uptake and drug diffusion. Higher crystallinity, as observed in HMW-PLLA and HMW-PCL, is associated with lower water permeability and slower drug release.

The plateau in release profiles is hypothesized to be a result of the interplay between drug diffusion and polymer degradation. Initially, the release is supposed to be diffusion-driven through water-filled pores, whereas the second phase corresponds to the release of the drug remaining trapped within the polymer matrix. Since polymer degradation is slow, this trapped drug remains unavailable, leading to incomplete release within the study timeframe. Thus, the release kinetics reflect both initial diffusion and delayed drug release associated with polymer degradation [45].

Although the release profile of HMW-PLLA might be considered aligned with the targeted release timeframe (6–9 months), the plateau observed after 20 weeks led to an incomplete drug release (30% of the total CLX released (Fig. 1)). This incomplete release was attributed to limited polymer degradation, which could lead to prototype limitations such as drug recrystallization [42]. Although recrystallization was not observed or specifically analyzed in this study, prolonged entrapment of drug molecules within the polymer matrix under aqueous conditions is generally considered a risk factor for potential recrystallization in drug delivery systems [46].

**Fig. 2:**
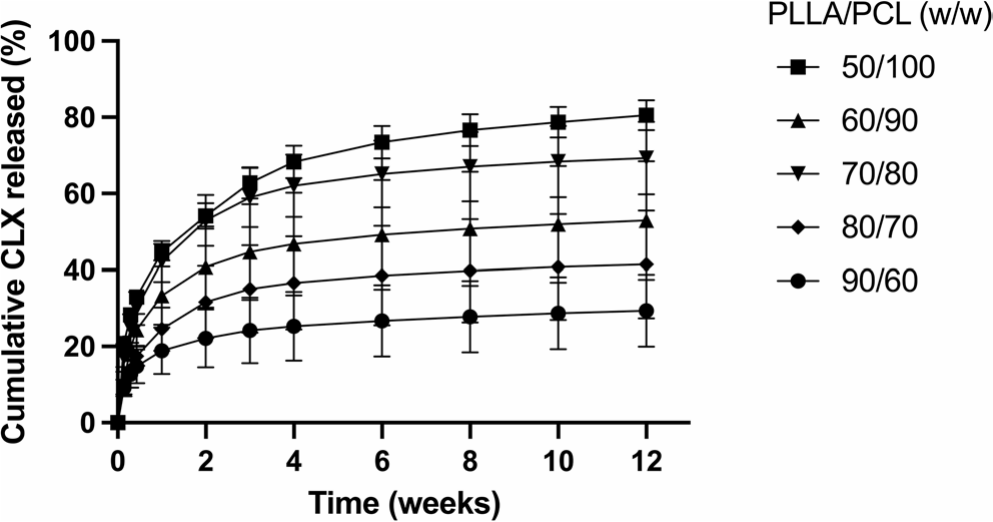
Release kinetics of CLX expressed as the total percentage of drug released (%) from PLLA/PCL blends prepared at 150 mg/mL at different (w/w) ratios (ranging from 50/100 to 90/60) with a CLX loading of 16.6%. **Abbreviations**: CLX: Celecoxib. PLLA: poly(L-lactide). PCL: poly(caprolactone). (w/w): weight-to-weight ratio. Values represent the mean ± standard deviation (*n* = 3)

To achieve intermediate CLX release kinetics, PLLA and PCL were blended at varying ratios (Fig. 2). All w/w ratios are expressed relative to a total polymer concentration of 150 mg/mL, which corresponds to the actual formulation conditions used for casting. Increasing PCL content accelerated CLX release, a trend attributed to the formation of polymer-to-polymer interfaces that facilitated water penetration and diffusion of the drug near these interfaces [47–50]. Due to the limited miscibility of PLLA and PCL, PCL domains aggregate, lowering Tg locally and increasing chain mobility. This facilitates nucleation and improves PLLA’s mechanical properties, including flexibility and toughness [51]. Among tested formulations, both PLLA/PCL blends at 70/80 (w/w) and 80/70 (w/w) showed promising results with intermediate release profiles among the blends studied, releasing 40% of CLX after 3 months without plateauing.

Given the variability in intra-articular (IA) CLX dosing across animal models (1-1.25 mg CLX/kg in mice [52], 0.46 mg CLX/kg/week over 5 weeks in rabbits [53], 0.0292 mg CLX/kg [54] or 1 mg/kg [55] in rats, and approximately 1 mg/kg in sheep [56]), it was assumed that higher doses of CLX could enhance therapeutic effects in both in vitro and in vivo models (e.g., reduced fibrous encapsulation, foreign body reactions (FBR), and modulation of signaling pathways). Hence, CLX concentration in selected formulations was increased 2.4-fold (from 12.5 mg/mL to 30 mg/mL), achieving a drug loading (DL) of 16.6%. This increase aimed to enhance the total amount of drug incorporated into the coating, bringing it closer to the IA doses previously reported in the ovine model. The PLLA/PCL blend at an 80/70 (w/w) ratio was ultimately selected for its balanced release profile (Fig. 2).

These results led to two emerging candidates for sustained CLX release: HMW-PLLA with 20% DL, which provided a very slow release (< 25% CLX released after 6 months), and PLLA/PCL 80/70 (w/w) with 16.67% DL, which achieved ~ 40% release in 3 months. Both formulations exhibited polymer-drug interactions based on thermal analysis (reductions in Tg, Tm, and Tcc) (Supplementary Fig. 3A) and successfully incorporated CLX in a molecularly dispersed form, as confirmed by the absence of CLX crystalline peaks in XRD and the disappearance of its melting endotherm peak in DSC thermograms (Supplementary Figs. 3A–B), both indicative of an amorphous drug state within the polymer matrix. However, only the PLLA/PCL 80/70 (w/w) blend met the required release profile and progressed to further studies.

#### DEX-loaded polymer films

To effectively address post-surgical acute inflammation, DEX was selected for its potent and pleiotropic anti-inflammatory activity and established clinical use. The goal was to achieve a rapid and controlled release over a 1 to 4-week period by incorporating DEX into an external polymer layer made of PLGA. A high molecular weight (MW) (24–38 kDa) was initially explored, but presented limitations due to its characteristic biphasic release profile (Supplementary Fig. 4) —marked by an initial burst from surface-associated drug, followed by a less predictable sustained release phase driven by bulk polymer hydrolysis [57, 58]. The degradation rate of PLGA decreases with increasing lactide content; however, the 50:50 lactide: glycolide ratio used in this study represents the fastest-degrading composition due to its high hydrophilicity and low crystallinity [59].

**Fig. 3:**
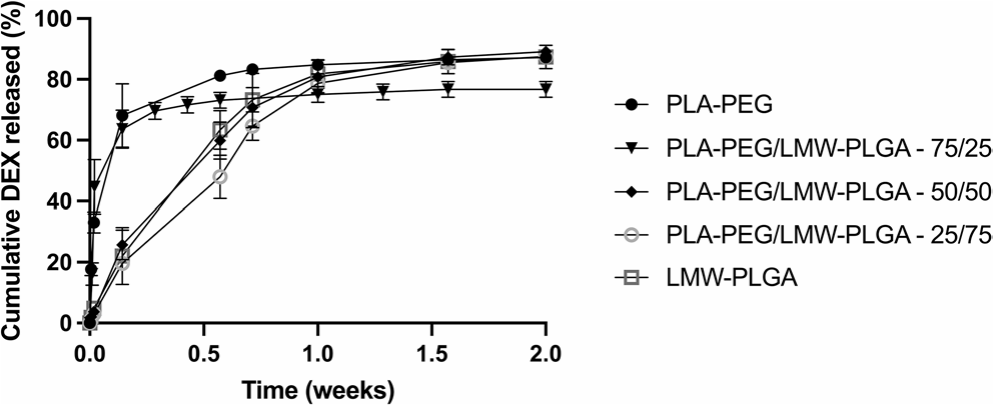
Release kinetics of DEX expressed as the total percentage of drug released (%) from pure PLA-PEG, pure LMW-PLGA, and PLA-PEG/PLGA blend films. LMW-PLGA was incorporated to the PLA-PEG matrix with different PLA-PEG/PLGA ratios ranging from 25/75 to 75/25 (w/w). Films were prepared at 200 mg/mL with constant drug loadings of 2.44%. Abbreviations: DEX: Dexamethasone. PLA-PEG: poly(lactic acid)-poly(ethylene glycol) di-block co-polymer. PLGA: Poly(lactic-co-glycolic) acid. LMW: Low molecular weight. Values represent the mean ± standard deviation (*n* = 3)

Given the limitations of PLGA, particularly its biphasic release profile, an alternative amphiphilic matrix—poly(lactic acid)-poly(ethylene glycol) di-block copolymer (PLA-PEG) (PEG = 5,000 Da)—was evaluated. Di-block copolymers like PLA-PEG can influence polymer-drug interactions differently than blends of individual polymers [60, 61] due to the covalent linkage between the blocks, which creates a more defined and stable microphase separation compared to physical blends [62]. This structural organization can alter drug distribution and water accessibility, ultimately affecting drug release kinetics and solubility. For these reasons, di-block copolymers have been widely used in drug delivery systems [63]. As shown in Fig. 3, PLA-PEG enabled a sustained and more complete release of DEX for approximately one week at a polymer concentration of 200 mg/mL. This accelerated release was likely due to PEG-induced aqueous swelling and the proximity of DEX within the matrix. However, the slow degradation rate of the PLA block could prolong the integrity of the top layer, acting as a persistent barrier over the bottom CLX-releasing layer and thereby limiting CLX diffusion to the release medium over time. To improve control over the release profile and mitigate the limitations associated with PLGA’s biphasic behavior, the effect of PLGA molecular weight (MW) on DEX release was investigated. In addition to the high-MW PLGA used initially, low-MW (LMW) variants (8 kDa) were tested both as standalone matrices and in blends with PLA-PEG (Fig. 3).

Blends of PLA-PEG and LMW-PLGA 75/25 (w/w) showed release profiles similar to pure PLA-PEG, but a 50/50 (w/w) blend extended the sustained DEX release to two weeks (Fig. 3). Increasing the relative amount of LMW-PLGA further enhanced this effect. Optimization studies with different PLA-PEG/LMW-PLGA ratios confirmed that higher LMW-PLGA content produced a sustained release within the desired 1–4-week window. Notably, pure LMW-PLGA exhibited a similar release profile, making it a practical alternative to PLA-PEG. Its selection not only met the therapeutic release target but also simplified the formulation by avoiding polymer blends.

DSC and XRD analyses were performed to confirm the physical state of the incorporated drugs. For both DEX and CLX formulations, the disappearance of their characteristic melting peaks in DSC thermograms and the absence of crystalline reflections in XRD patterns confirmed that the drugs were molecularly dispersed within the polymer matrices (Supplementary Figs. 3A and B, and 5A and B). For CLX-loaded systems, thermal events and crystallinity indicated good miscibility and a predominantly amorphous dispersion of the drug. Slight reductions in Tg and Tm—particularly in PLLA-based formulations—suggested plasticizing effects and increased polymer chain mobility [47, 64–66]. These interactions are consistent with the initial diffusion-driven release observed in PLLA and PLLA/PCL blends. Notably, no direct correlation was found between polymer crystallinity and release kinetics, supporting the idea that other factors—such as porosity and polymer-drug interactions—played a more dominant role [67]. In the case of DEX, minor increases in crystallinity were observed in PEG-containing systems, suggesting weak drug-polymer interactions that may facilitate water uptake and diffusion. Conversely, PLGA and LMW-PLGA matrices remained largely amorphous, aligning with their more sustained release behavior. A full list of thermal parameters (Tg, Tm, Xc) is provided in Supplementary Tables 1 and 2.

This comprehensive screening identified PLLA/PCL (80/70, w/w) with 16.6% DL as the most promising formulation for sustained CLX release, achieving higher drug incorporation while avoiding the plateau effect observed with pure PLLA. For DEX release, both PLA-PEG and LMW-PLGA (8 kDa, 50:50 LA: GA), each with a DL of 2.44%, achieved controlled release within the target 1–4 week window, with LMW-PLGA providing a slightly more sustained release profile and the advantage of a simpler formulation.

### Engineering a bilayer drug-releasing polymer coating for the sequential release of DEX and CLX

To enable the simultaneous release of CLX and DEX from the non-biodegradable PCU prosthesis (~ 0.7 × 0.7 × 0.3 cm), a bilayer polymeric coating was developed. The system consists of a first layer releasing CLX to be cast on top of the prosthesis, and a second layer releasing DEX, providing a shorter drug release. Polymer matrices previously identified as optimal candidates (see section Design and optimization of drug-loaded polymer films) were selected for testing in this configuration. While solvent casting was effective for coating one surface, dip coating was chosen for full-implant coverage due to its simplicity, versatility, and compatibility with multilayer deposition [18]. This section explores key aspects of bilayer development, including the impact of solvent interactions between layers and the selection of one optimized polymer matrix for each drug for final implementation.

Given that the DEX layer is prepared using acetone, the potential effect of solvent exposure on the underlying CLX-loaded layer was evaluated. Since acetone can partially disrupt polymer-drug interactions, this investigation was crucial for ensuring stability and consistency in release kinetics. This was explored by DSC, XRD, and drug release evaluation— detailed in Supplementary Figs. 6 and 7. When acetone was applied to PLLA/PCL films, a slight acceleration in CLX release was noted, yet the overall release kinetics remained consistent. However, for pure PLLA, acetone significantly altered the release profile, accelerating CLX release from approximately 10% to over 60% by week 21 (Supplementary Fig. 7).

#### Evaluation of drug release

**Fig. 4:**
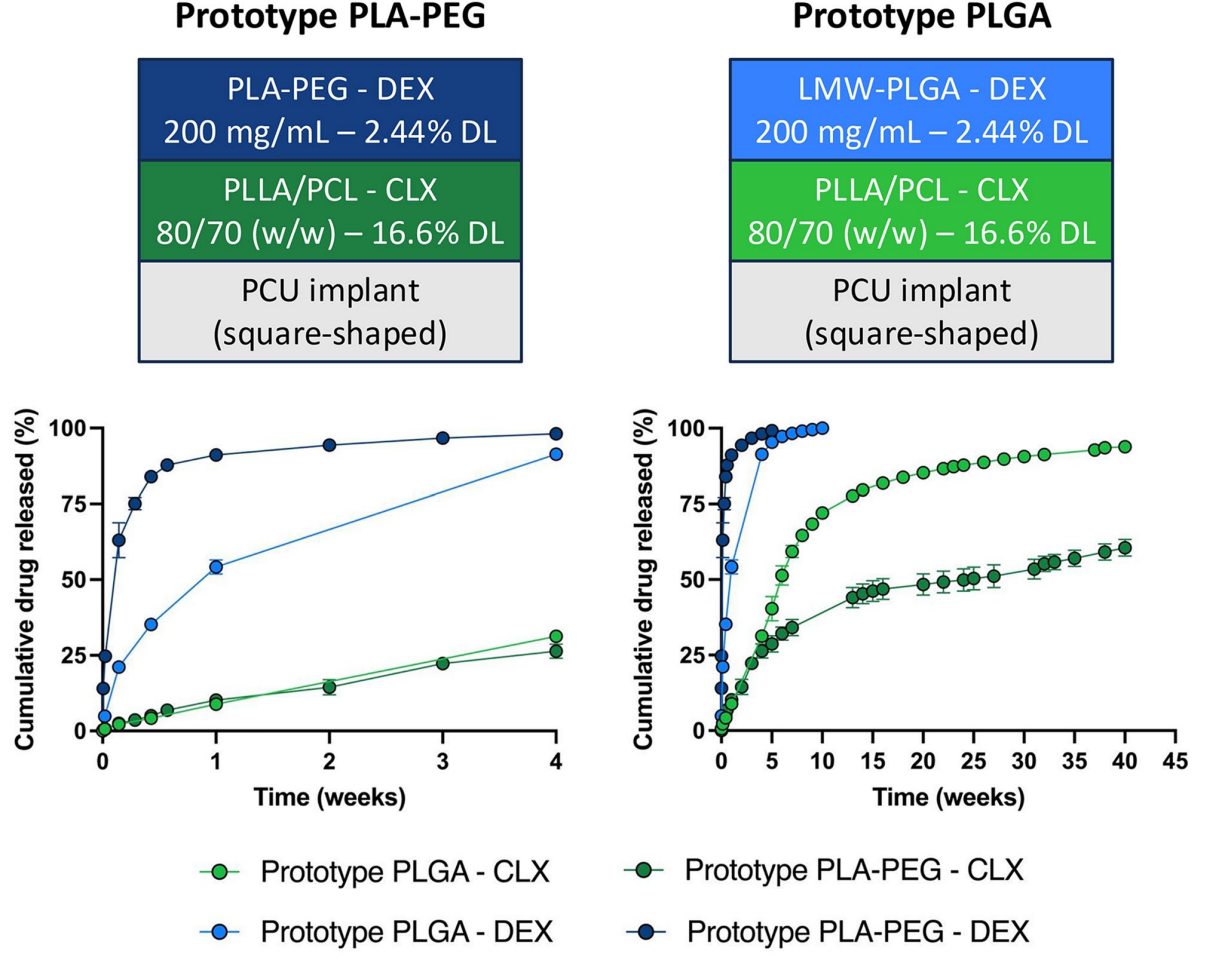
Schematic representation of the two final prototypes of bilayer polymer coatings **(A)**. Sequential release of DEX and CLX from bilayer polymer coatings composed of a first polymer coating of PLLA/PCL, prepared at 150 mg/mL at 80/70 (w/w) with CLX loading of 16.6%; and a second polymer coating of either PLA-PEG or LMW-PLGA, both prepared at 200 mg/mL with DEX loading of 2.44% **(B)**. Abbreviations: PCU: Polycarbonate urethane. CLX: Celecoxib. DEX: Dexamethasone. PLA-PEG: poly(lactic acid)-poly(ethylene glycol) di-block co-polymer. PLGA: Poly(lactic-co-glycolic) acid. PLLA: poly(L-lactide). PCL: poly(caprolactone). LMW: Low molecular weight. Values represent the mean ± standard deviation (*n* = 3). Both prototypes share the same CLX-releasing bottom layer (PLLA/PCL), while the top layer differs and carries DEX (PLA-PEG or LMW-PLGA)

The release profiles of DEX from the two polymer matrices—PLA-PEG and LMW-PLGA— were evaluated while maintaining a constant CLX-releasing bottom layer composed of the PLLA/PCL blend. PCU implants were coated using a dip-coating approach, where CLX was first incorporated into the PLLA/PCL film using DCM, and DEX was then incorporated into either PLA-PEG or LMW-PLGA using acetone. The main distinction between these bilayer systems lies in the choice of the top-layer polymer, which influenced not only DEX release but also indirectly modulated CLX release kinetics. For clarity, these systems are referred to as Prototype PLA-PEG and Prototype PLGA (Fig. 4A). Together, they provide valuable design insights for dual-drug delivery strategies targeting both acute and chronic phases of inflammation.

Despite the identical CLX-releasing layer, the release profiles of CLX varied depending on the top-layer polymer (Fig. 4B). When LMW-PLGA was used as the top layer, CLX release was faster, likely because the lower molecular weight and higher hydrophilicity of the PLGA facilitated water uptake and swelling, which enhanced buffer infiltration into the underlying CLX-loaded layer. Although both LMW-PLGA and PLA-PEG are capable of water uptake and swelling, the impact of this property on drug release depends on the system architecture. In single-layer matrices with embedded DEX, swelling enhances release directly by facilitating diffusion, thus PLA-PEG showed the fastest release of DEX. In contrast, in the bilayer system, the top-layer swelling modulates access to the underlying drug reservoir, making both the permeability and molecular weight of the top layer critical to the overall release kinetics. As a result, LMW-PLGA as the top layer led to faster release of CLX from the bottom layer. Furthermore, the slower-degrading PLA-PEG formed a more persistent barrier, delaying access of the release medium to the CLX layer and thus slowing its diffusion. This interpretation aligns with previous reports describing swelling-mediated release mechanisms in low-MW polymers [44].

DEX release profiles were consistent with our prior results, where PLA-PEG exhibited an initial burst followed by a gradual tapering, while LMW-PLGA provided a more sustained and steady release. Both systems achieved complete DEX release within the first few weeks, effectively meeting the goal of addressing acute post-surgical inflammation.

#### In vitro degradation of PLA-PEG and PLGA bilayer coated implants

Although both bilayer prototypes (PLA-PEG and PLGA) achieved the desired release kinetics for DEX and CLX, they exhibited distinct release profiles. Thus, using these prototypes, degradation studies were conducted to better understand the mechanisms behind their different release behaviors and to validate the hypothesis that PLA-PEG degrades more slowly than PLGA. Coated square implants were immersed in PBS supplemented with 1% (w/v) Tween^®^80, and periodically analyzed for trends in the change of pH and thickness, as well as surface morphology via FESEM.

**Fig. 5:**
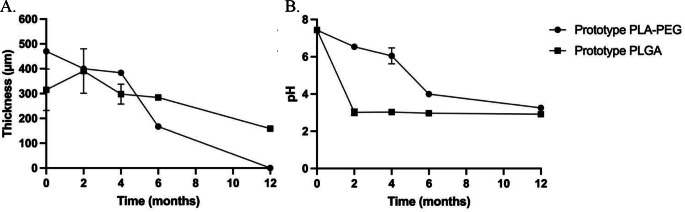
Changes in thickness **(A)** and pH **(B)** of Prototype PLA-PEG and Prototype PLGA with time. Abbreviations: PLA-PEG: poly(lactic acid)-poly(ethylene glycol) di-block co-polymer. PLLA: poly(L-lactide). PCL: poly(caprolactone). PLGA: Poly(lactic-co-glycolic) acid. µm: micrometers. Values represent the mean ± standard deviation (*n* ≥ 1)

**Fig. 6:**
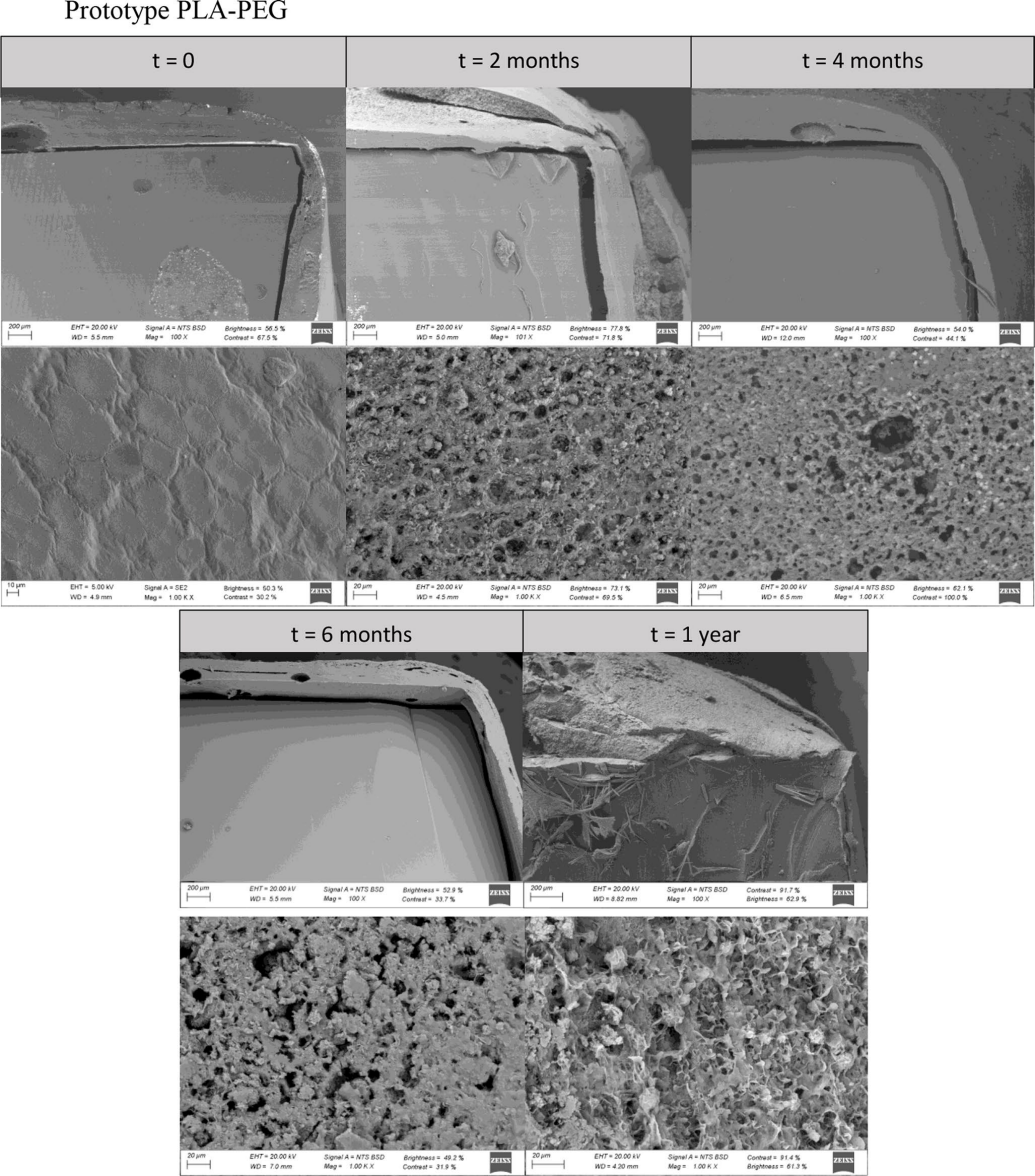
FESEM images of the Prototype PLA-PEG at different times of the biodegradation process. Images are divided into pairs per time point, a top image and a bottom image. The top images correspond with the sagittal cut of the bilayer polymer coating and the PCU implant and were obtained using Zeiss EVO analytical FESEM with a magnification of 100X. The thickness of the polymer coating was measured in these top images. The bottom images correspond with the view from above of the bilayer polymer coating sputter coated with iridium and were obtained using Zeiss UltraPlus analytical FESEM with a magnification of 1000X. Abbreviations: FESEM: Field emission scanning electron microscopy. PLA-PEG: poly(lactic acid)-poly(ethylene glycol) di-block co-polymer. µm: micrometers. EHT: Electron high tension. WD: Working distance. Mag: Magnification

**Fig. 7:**
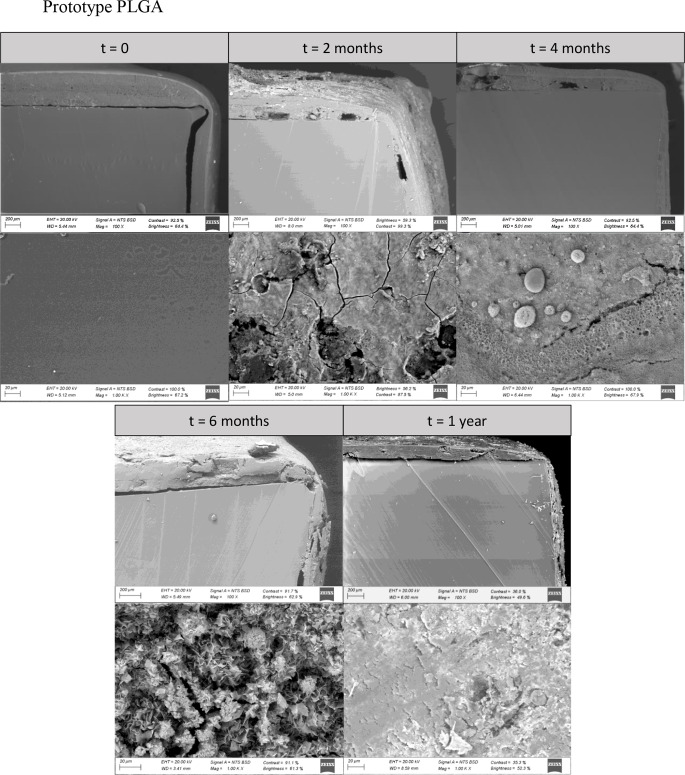
FESEM images of the Prototype PLGA at different times of the biodegradation process. Images are divided into pairs per time point, a top image and a bottom image. The top images correspond with the sagittal cut of the bilayer polymer coating and the PCU implant and were obtained using Zeiss EVO analytical FESEM with a magnification of 100X. The thickness of the polymer coating was measured in these top images. The bottom images correspond with the view from above of the bilayer polymer coating sputter-coated with iridium and were obtained using Zeiss UltraPlus analytical FESEM with a magnification of 1000X. Abbreviations: FESEM: Field emission scanning electron microscopy. PLGA: Poly(lactic-co-glycolic) acid. µm: micrometers. EHT: Electron high tension. WD: Working distance. Mag: Magnification

Over time, Prototype PLA-PEG showed a gradual decrease in thickness, with a pronounced reduction between 4 and 6 months, corresponding to clear structural degradation and coating disintegration (Figs. 5A and 6). By 12 months, the coating was visibly deteriorated, appearing uneven and incomplete, with remarkable signs of degradation and areas where the prosthesis surface was no longer covered. In contrast, Prototype PLGA initially exhibited a thickness increase—likely due to polymer swelling—followed by a steady decrease over the year (Figs. 5A and 7) [68, 69]. The pH monitoring unveiled additional aspects of the degradation behavior not apparent from thickness measurements alone. While Prototype PLA-PEG maintained relatively stable pH values for the first 4 months, a noticeable decline occurred between months 4 and 6, consistent with the delayed onset of degradation observed in FESEM analysis and expected of PLA. In contrast, Prototype PLGA triggered a sharp pH drop by month 2, which then remained low for the rest of the study (Fig. 5B). These trends reflect the faster breakdown of the PLGA matrix and further support the interpretation that the top-layer degradation rate directly influenced buffer access to the CLX-releasing layer.

These findings help explain the differences observed in CLX release profiles. Although both prototypes had the same PLLA/PCL internal layer for CLX release, faster degradation and reduced tortuosity of the PLGA top layer likely allowed earlier buffer access to the underlying CLX matrix, accelerating drug release. In contrast, the slower degradation of PLA-PEG maintained a more effective diffusion barrier for longer. Thickness measurements were affected by sample cutting and imaging variability because of the angle of the image and should be interpreted as relative trends rather than absolute values (Supplementary Fig. 8). In contrast, pH changes provided a more robust and reproducible indicator of degradation, though results may not fully reflect in vivo behavior, where pH buffering and enzymatic processes differ. Nonetheless, prior work suggests that PLGA degradation kinetics in vitro correlate with in vivo molecular weight loss [134].

#### Evaluation of drug-releasing polymer coating reproducibility

The reproducibility of the prototypes was assessed by quantifying the total amount of DEX and CLX per unit of area (µg/cm²) across multiple replicates of square-shaped PCU implants. The Prototype PLGA showed consistent DL for both DEX and CLX, indicating a robust and reproducible coating process with controlled dipping cycles and solvent evaporation. In contrast, Prototype PLA-PEG showed slight variability in drug incorporation between replicates, suggesting challenges in achieving uniform deposition. This may be attributed to uneven polymer film formation, variations in drug-polymer interactions, or greater sensitivity to coating conditions on the complex implant surface. These results indicate that Prototype PLGA offers greater reliability and process reproducibility, while Prototype PLA-PEG may require further optimization to ensure consistent DL (Supplementary Fig. 9).

### In vitro assessment of sterility, biocompatibility, anti-inflammatory activity, and efficiency of the bilayer drug-releasing polymer-coated PCU implants

Prior to evaluating the immunomodulatory potential of the bilayer-coated implants, a series of in vitro validations were conducted to ensure compatibility with biological assays. Drug release studies confirmed that both DEX and CLX were released in concentrations exceeding their respective effective thresholds (10 µM for DEX, 25 µM for CLX, based on literature [26]) at key time points—3 h, 3 days, 1 week, 2 weeks, and 4 weeks (Table 1). These intervals were strategically selected to capture distinct drug activity phases: early DEX-driven effects, overlapping DEX and CLX activity, and later CLX-dominant responses.

The release buffer also met sterility criteria, with endotoxin levels remaining below 0.125 EU/mL (Supplementary Fig. 10), validating their suitability for macrophage-based assays. Moreover, no cytotoxic effects were observed in HMDMs exposed to release media from either prototype at any time point, indicating good biocompatibility of both the drugs released and the polymer degradation byproducts (Supplementary Fig. 11). These findings are consistent with previous reports describing the non-toxic nature of polyester degradation products and further support the safety of the materials used [70]. With drug release, sterility, and cytocompatibility confirmed, the subsequent experiments focused on assessing the bioactivity of the released drugs by evaluating their capacity to modulate cytokine secretion in M1-like (classically activated with LPS + IFNγ) pro-inflammatory macrophages.

#### In vitro evaluation of Immunomodulatory activity

The evaluation of cytokine secretion aimed to confirm that the drugs released from the bilayer polymer coatings retained their immunomodulatory activity. Specifically, it was sought to verify that DEX and CLX, when released at their respective time points, could suppress pro-inflammatory cytokine release from macrophages, events associated with acute and chronic inflammation. This assessment was essential to validate the therapeutic functionality of the coating system in an inflammatory environment. Cytokine secretion by M1-like macrophages was measured following exposure to release media collected from the implant incubations at defined time points. The three key pro-inflammatory cytokines selected were TNF-α (a central mediator of acute inflammation [71–73]), CCL2 (MCP-1) (regulates monocyte/macrophage recruitment and foreign body giant cell (FBGC) formation [74, 75]), and PGE2 (a COX-2 pathway product involved in joint inflammation, vasodilation, and pain [76, 77]).

**Fig. 8:**
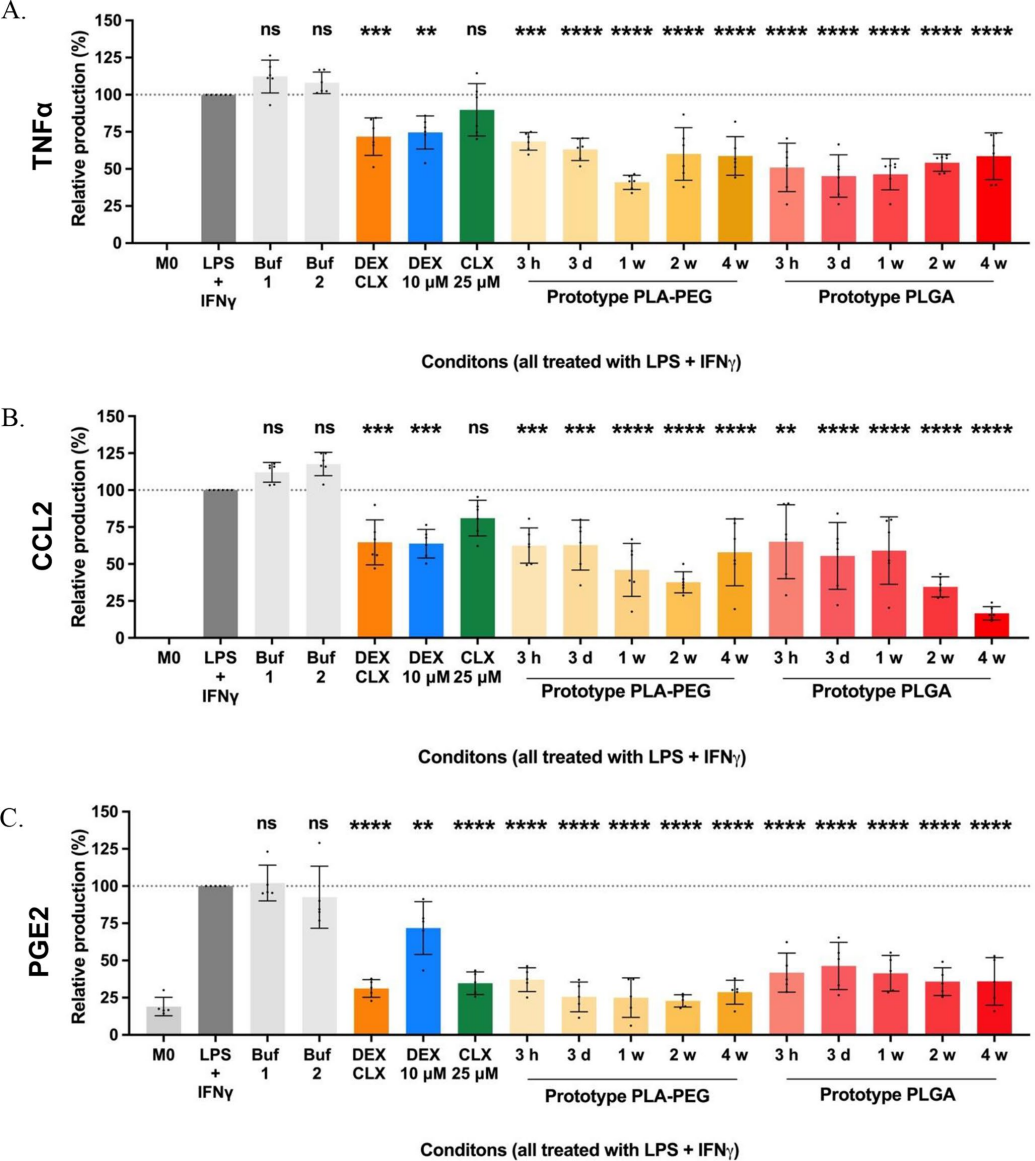
TNFα **(A)**, CCL2 **(B)**, and PGE2 **(C)** secretion by human primary macrophages exposed to media where Prototype PLA-PEG and Prototype PLGA, both loaded with CLX and DEX, were incubated for specific periods of time. Analysis was carried out by ELISA. Abbreviations: M0: Unactivated macrophages. Neg: Negative. Pos: Positive. Buf: Buffer. PLA-PEG: poly(lactic acid)-poly(ethylene glycol) di-block co-polymer. h: hour. d: days. w: weeks. PLGA: Poly(lactic-co-glycolic) acid. LPS: Lipopolysaccharide. IFN: Interferon. TNF: Tumor necrosis factor. CCL2: C-C motif chemokine ligand 2 or Monocyte chemoattractant protein-1 (MCP-1). PGE2: Prostaglandin E2. A significant comparison was performed using an ordinary one-way ANOVA followed by Tukey’s multiple comparison tests between LPS + IFNy and the rest of the groups. p-values < 0.05 were considered statistically significant (*). Also, (**) if *p*-value < 0.01, (***) if *p*-value < 0.001, (****) if *p*-value < 0.0001. ns: not significative. Columns represent the mean ± standard deviation (*n* ≥ 5)

Both prototypes significantly suppressed TNF-α and CCL2 secretion across all time points (Fig. 8A and B), demonstrating that the combination of DEX and CLX maintained its anti-inflammatory efficacy following their release from the coatings. Notably, TNF-α and CCL2 suppression was more closely linked to DEX activity, as expected during early time points. PGE2 secretion, in contrast, was more selectively reduced by CLX, consistent with its inhibition of COX-2 (Fig. 8C) [78]. This effect was observed at all time points, regardless of the prototype, supporting the stability and retained bioactivity of CLX even after extended residence within the coating. Importantly, cytokine suppression levels were comparable to those obtained with free (non-encapsulated) drugs, validating the effectiveness of the delivery platform. These results confirm that both Prototype PLGA and Prototype PLA-PEG effectively preserved the bioactivity of the drugs during encapsulation, storage, and release.

## Conclusions

Here we present a bilayer polymer coating developed to enable the sequential release of dexamethasone (DEX) and celecoxib (CLX), from a polycarbonate urethane (PCU) meniscus prosthesis, with the final goal of mitigating both, acute and chronic inflammation observed after implantation. Through a systematic screening of polymer candidates, two bilayer prototypes—Prototype PLA-PEG and Prototype PLGA—were identified based on their ability to achieve the desired release kinetics: rapid DEX release over 1–4 weeks to prevent acute inflammation, and prolonged CLX release over 6–9 months for sustained immunomodulation. Polymer degradation studies revealed that Prototype PLGA, composed of a low molecular weight PLGA external layer, exhibited faster degradation, facilitating earlier exposure of the CLX-loaded PLLA/PCL bottom layer to the release medium. In contrast, Prototype PLA-PEG was degraded more slowly, increasing tortuosity and delaying CLX release. Although PLA-PEG allowed for higher drug loafing, PLGA demonstrated more consistent and reproducible release profiles and better integration with the bilayer system. Both prototypes were endotoxin-free and biocompatible, and the released drugs maintained their anti-inflammatory activity, as demonstrated by the effective decrese in the secretion of pro-inflammatory cytokines by M1-like activated macrophages.

Based on its favorable degradation kinetics, reproducibility, and compatibility with the bilayer architecture, Prototype PLGA was selected for further development. This next phase would involve optimizing the coating system and scaling up its application to actual meniscus prostheses for evaluation in a representative animal model, bringing the technology closer to clinical translation.

## Supplementary Information

Below is the link to the electronic supplementary material.

Supplementary Material 1

## Data Availability

The datasets generated during and/or analysed during the current study are available from the corresponding author on reasonable request.
