# Supplementary material for: Controlled co-delivery of anti-inflammatory drugs from bilayer polymer films coating a meniscus implant
Source: Drug Deliv Transl Res. 2025 Aug 25;16(7):2207–25. doi: 10.1007/s13346-025-01942-5 (PMC13294237; doi:10.1007/s13346-025-01942-5)
Supplement: Supplementary file 1 — Supplementary Material 1 [file 13346_2025_1942_MOESM1_ESM.docx]

**Supplementary**


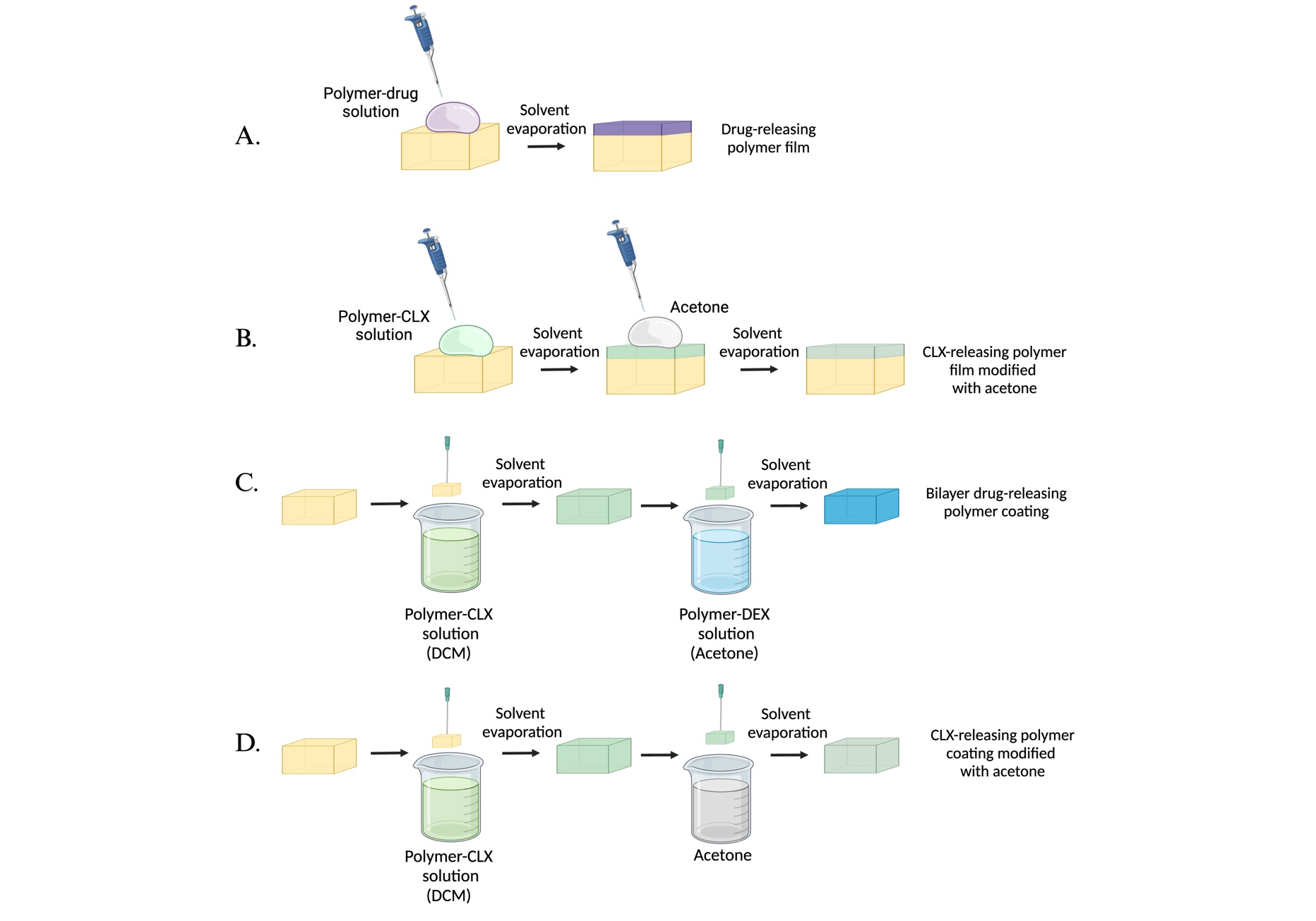


**Supplementary Figure 1. Schematic representation of solvent casting (A,B) and dip coating (C,D) techniques. Drug-releasing polymer films used for screening, DSC, and XRD (A,B). Bilayer drug-releasing polymer coatings used for screening (C,D). Drug-releasing polymer films or coatings modified with acetone to evaluate the effect of the organic solvent of the top layer on the bottom layer (B,D).**

**Abbreviations:** CLX: Celecoxib. DEX: Dexamethasone. DCM: Dichloromethane. Created with https://www.biorender.com/


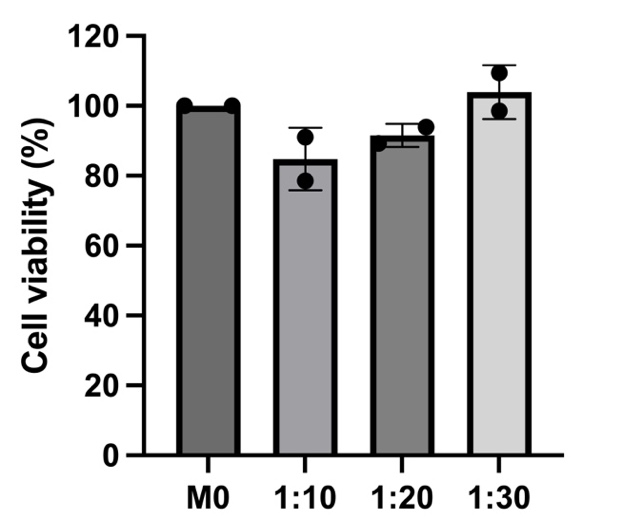


**Supplementary Figure 2. AlamarBlue™ cell viability assay showing the effects of different dilutions of the release media in RPMI on cell viability in vitro.**

**Abbreviations:** M0: Unactivated macrophages. Values represent the mean ± standard deviation (n=2).


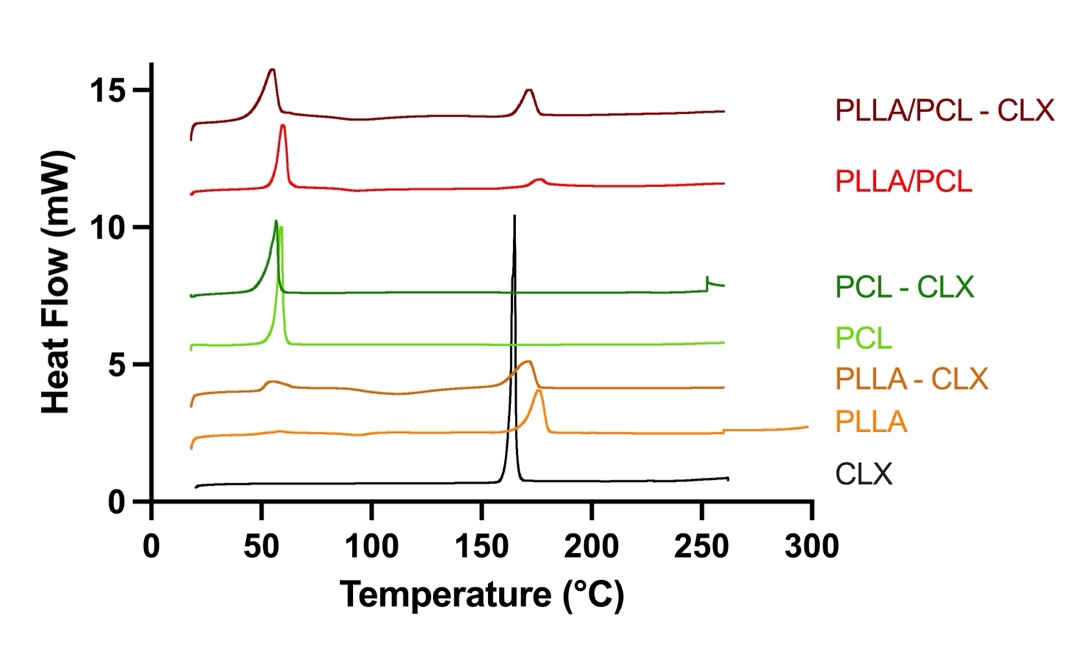


B.

A.


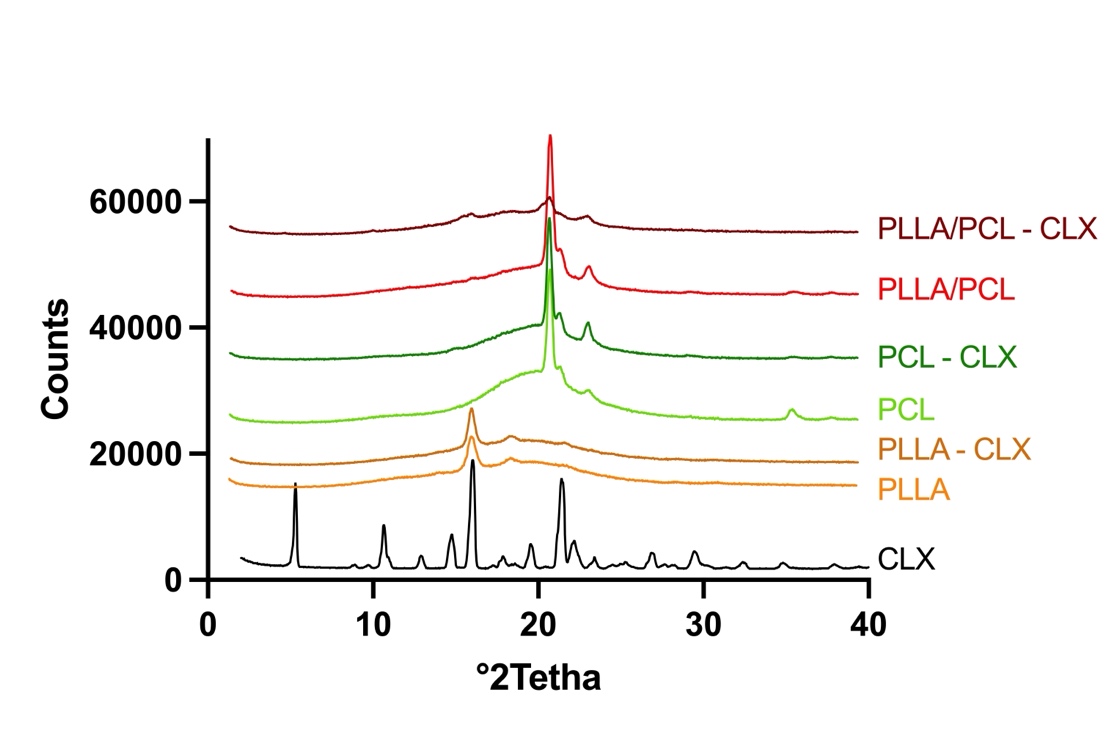


Supplementary Figure 3. *DSC thermograms (A) and XRD diffractograms (B) of the CLX-releasing prototypes.*

**Abbreviations:** CLX: Celecoxib. PLLA: poly(L-lactide). PCL: poly(caprolactone). mW: milli Watts.


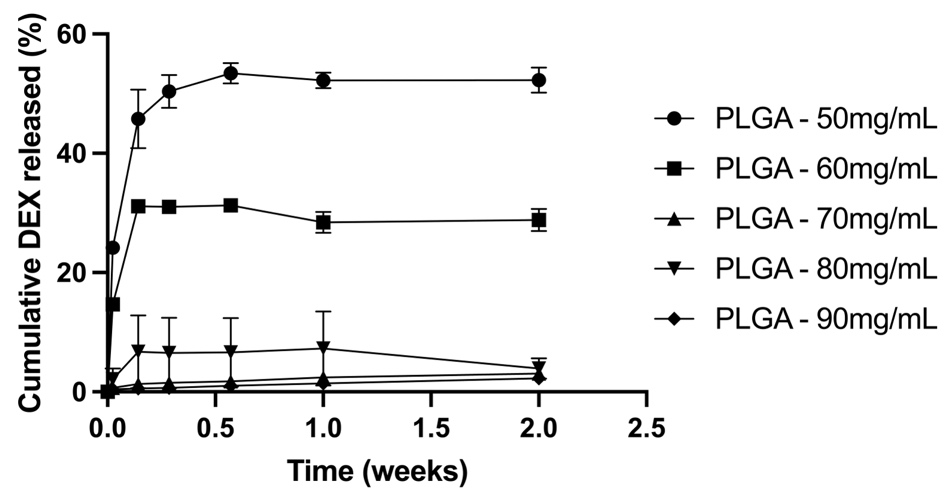


Supplementary Figure 4. Release kinetics of DEX expressed as the total percentage of drug released (%) from PLGA prepared at different concentrations ranging from 50 to 90 mg/mL with varying drug loadings but keeping the drug concentration constant at 5 mg/mL.

**Abbreviations:** DEX: Dexamethasone. PLGA: Poly(lactic-co-glycolic) acid. mg: milligrams. mL: milliliters. Values represent the mean ± standard deviation (n=3).

**
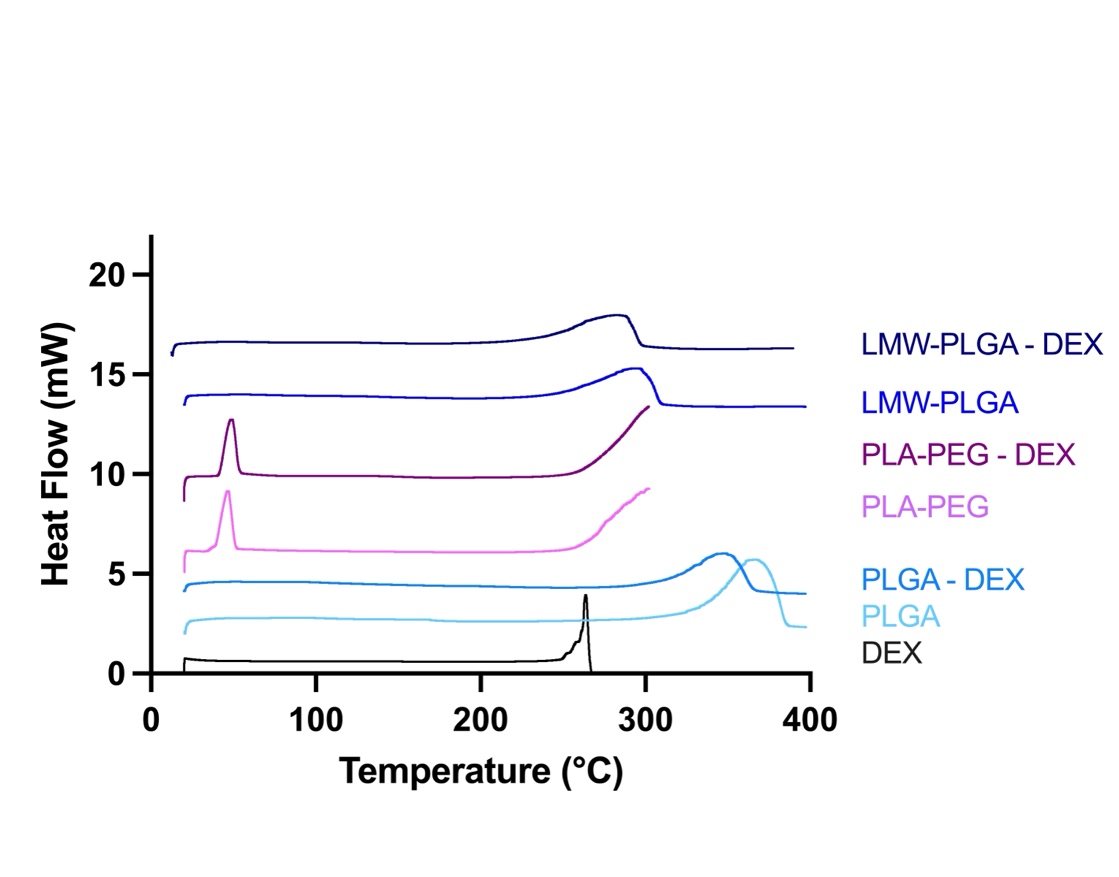
**

A.

B.

**
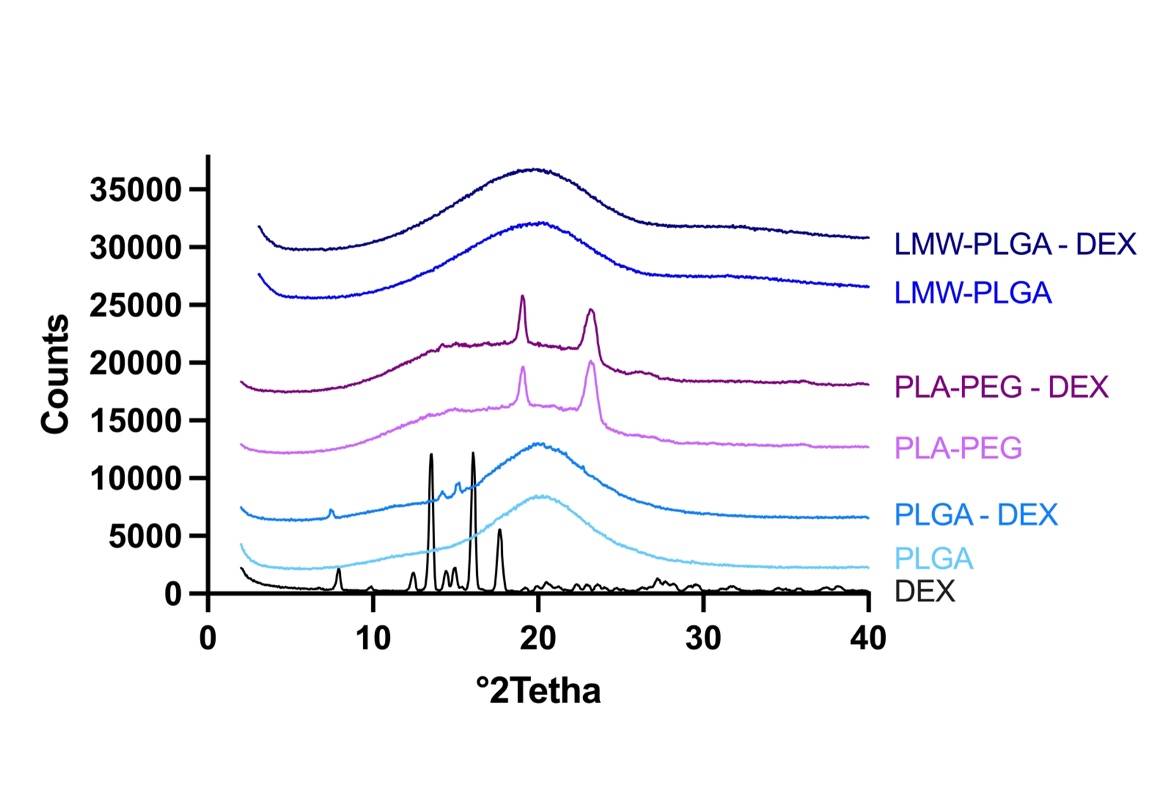
**

Supplementary Figure 5. *DSC thermograms (A) and XRD diffractograms (B) of the DEX-releasing prototypes.*

Abbreviations: DEX: Dexamethasone. PLGA: Poly(lactic-co-glycolic) acid. PLA-PEG: poly(lactic acid)-poly(ethylene glycol) di-block co-polymer. HMW: Low molecular weight. LMW: Low molecular weight. mW: milli Watts.

*
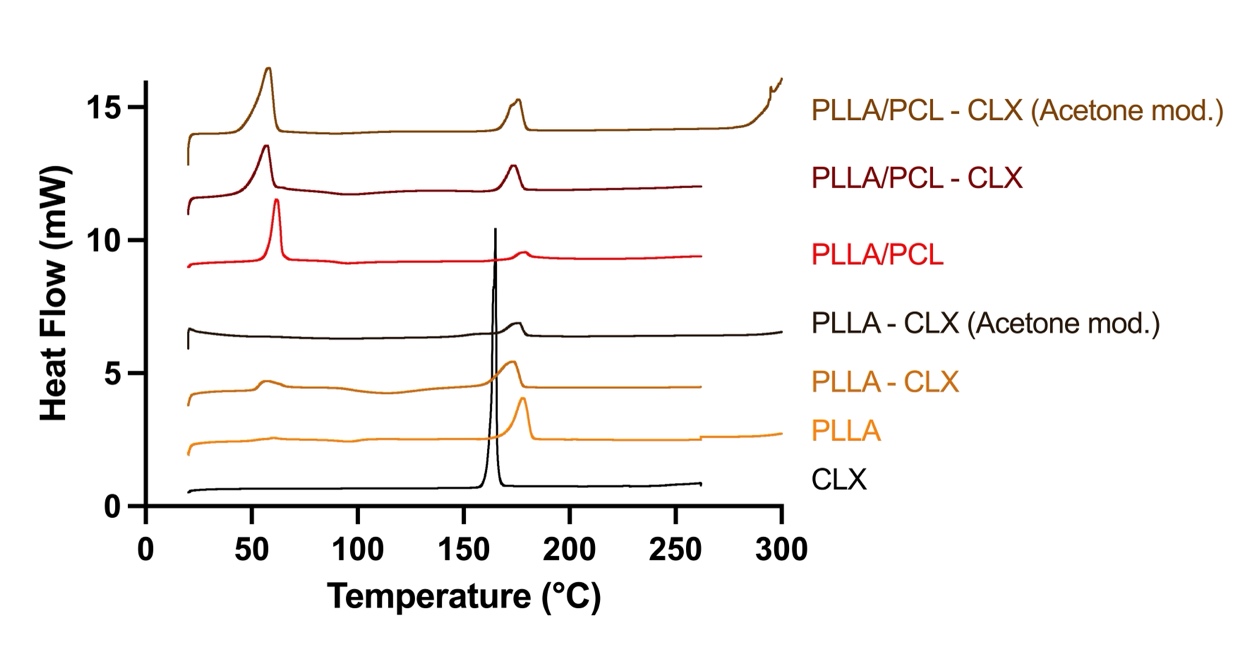
*

A.

*
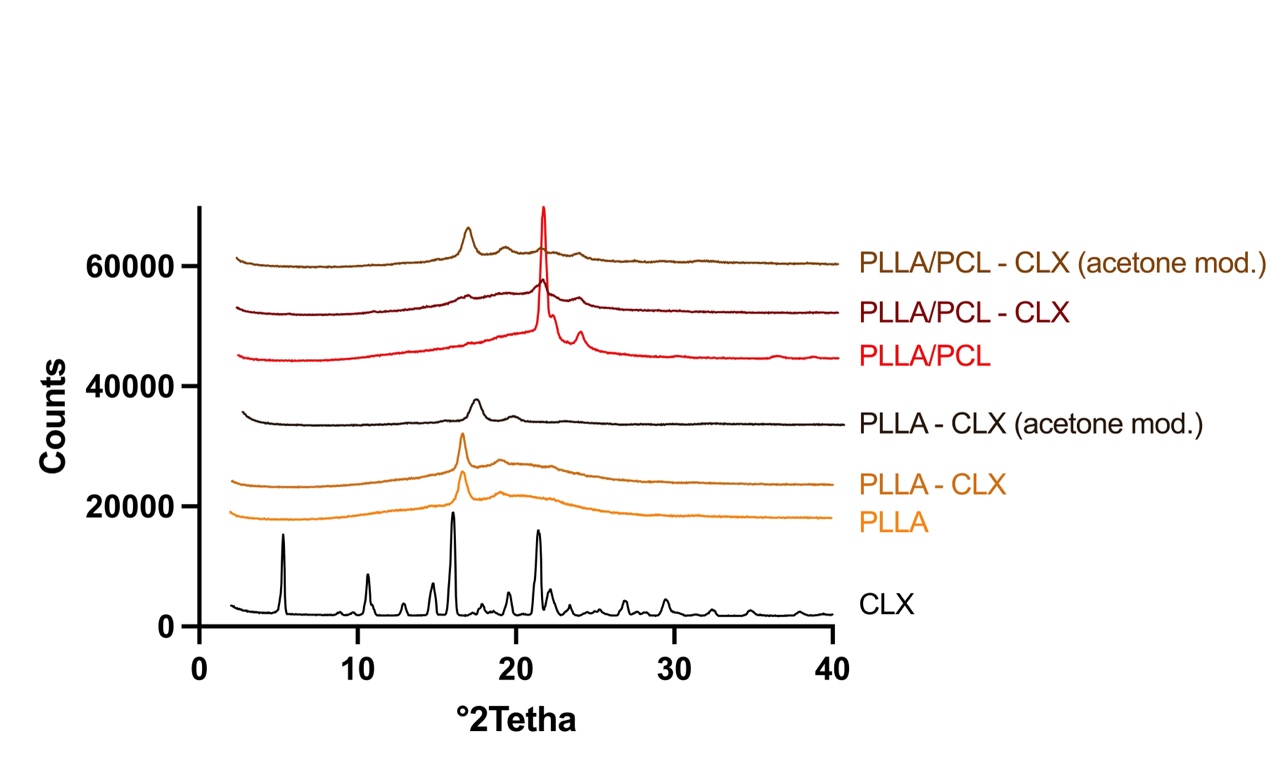
*

B.

Supplementary Figure 6. *DSC thermograms (A) and XRD diffractograms (B) of the acetone-modified CLX-releasing prototypes.*

**Abbreviations:** CLX: Celecoxib. PLLA: poly(L-lactide). PCL: poly(caprolactone). mod.: modified. mW: milli Watts.


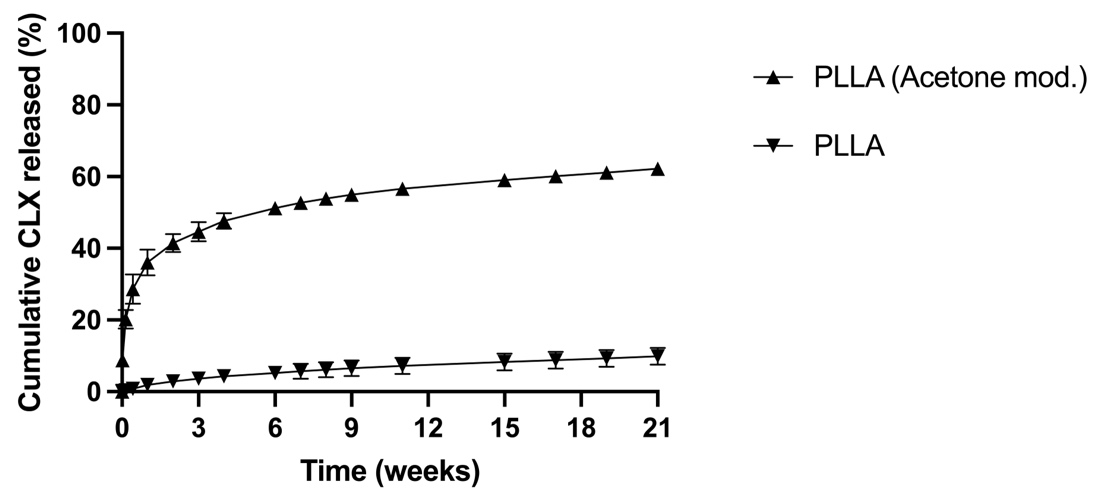

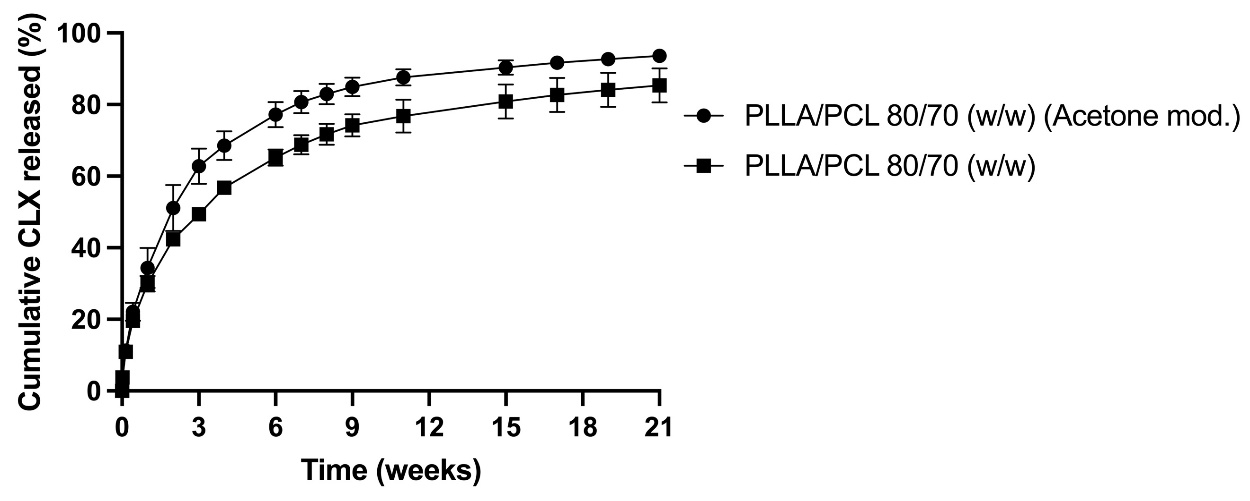


A.

B.

Supplementary Figure 7. Release kinetics of CLX expressed as the total percentage of drug released (%) from unmodified or acetone-modified PLLA prepared at 50 mg/mL with a CLX loading of 20% (A) and unmodified or acetone-modified PLLA/PCL blends prepared at 150 mg/mL at 80/70 (w/w) with a CLX loading of 16.67% (B).

Abbreviations: CLX: Celecoxib. PLLA: poly(L-lactide). PCL: poly(caprolactone). (w/w): weight to weight ratio. mod.: modified. Values represent the mean ± standard deviation (n=3).


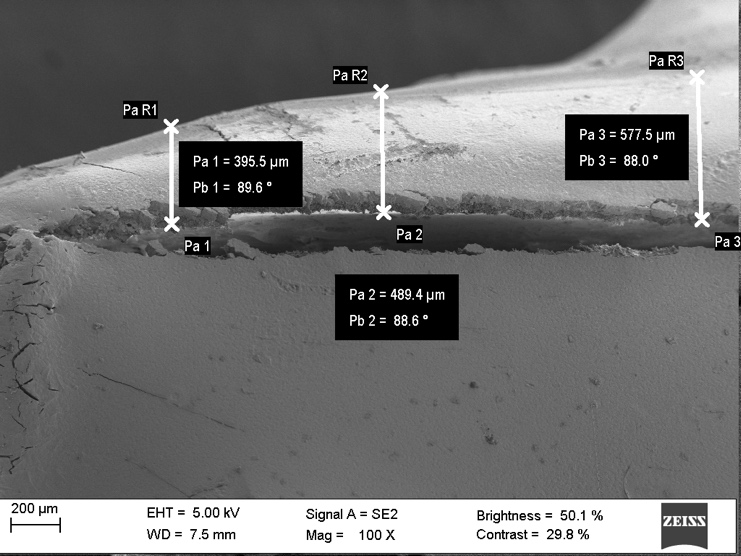


Supplementary Figure 8. Representative image of how the thickness of the polymer coatings was measured using FESEM (in this case, image corresponds with Prototype PLA-PEG at time 0 of the biodegradation process). The image corresponds with the sagittal cut of the bilayer polymer coating and the PCU implant and was obtained using Zeiss EVO analytical FESEM with a magnification of 100X.

**Abbreviations:** FESEM: Field emission scanning electron microscopy. PLGA: Poly(lactic-co-glycolic) acid. µm: micrometers. EHT: Electron high tension. WD: Working distance. Mag: Magnification.


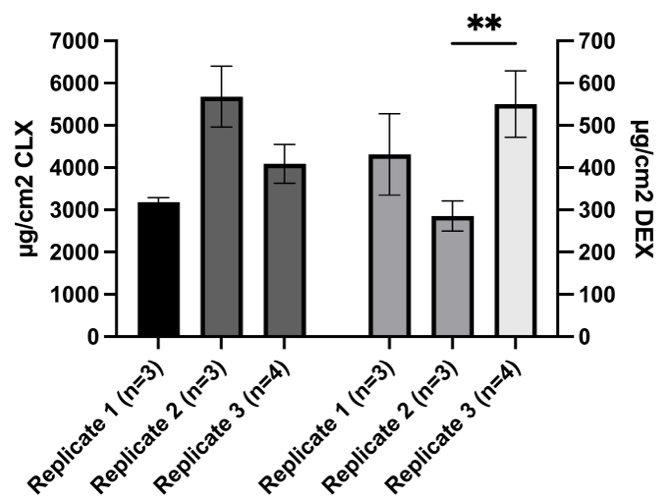

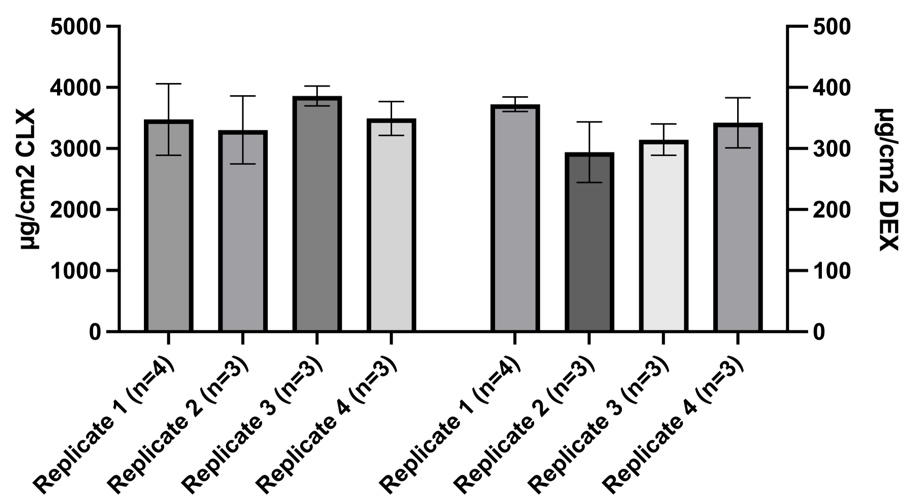


Supplementary Figure 9. DL of both CLX and DEX in Prototype PLA-PEG (A) and Prototype PLGA (B) across intra- and inter-day replicates.

**Abbreviations:** CLX: Celecoxib. DEX: Dexamethasone. µg: micrograms. cm^2^: square centimeters. A significant comparison was performed using a Brown-Forsythe and Welch one-way ANOVA followed by Tukey’s multiple comparison tests between groups. *p*-values < 0.05 were considered statistically significant (*). Also, (**) if *p*-value < 0.01. When no asterisk is displayed, not significative differences (ns) were observed. Columns represent the mean ± standard deviation (n ≥ 3). Each replicate corresponds with an inter-day replicate.


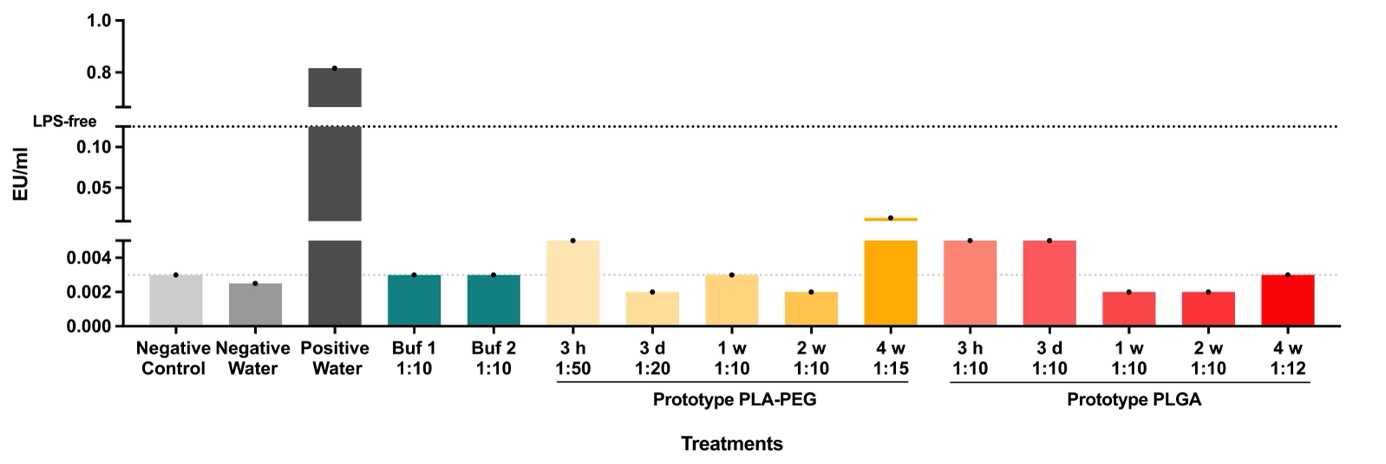


**Supplementary Figure 10. Chromogenic LAL-test to test the media, containing drugs, released from the Prototype PLA-PEG and Prototype PLGA at indicated times, to determine the endotoxin contamination prior to initiate the immunotoxicity evaluation.**

**Abbreviations:** EU: Endotoxin units. ml: milliliter. Neg: Negative. Pos: Positive. Buf: Buffer. PLA-PEG: poly(lactic acid)-poly(ethylene glycol) di-block co-polymer. h: hour. d: days. w: weeks. PLGA: Poly(lactic-co-glycolic) acid. Columns represent the mean ± standard deviation (n = 1).


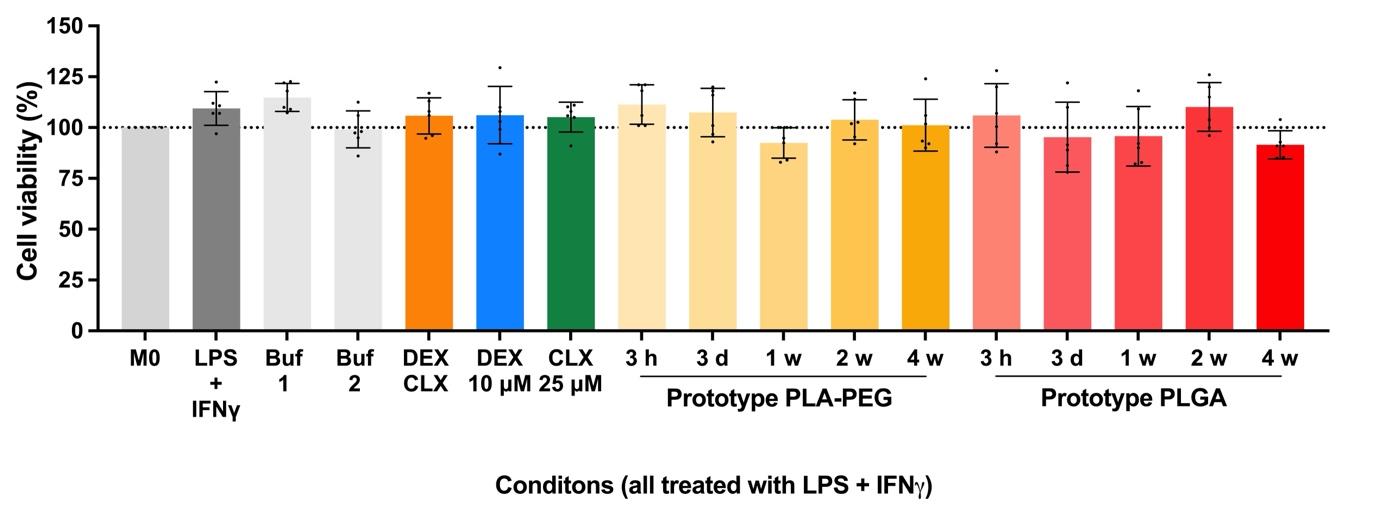


Supplementary Figure 11. AlamarBlue™ cell viability assay to evaluate the biocompatibility of Prototype PLA-PEG and Prototype PLGA at indicated times.

**Abbreviations:** M0: Unactivated macrophages. Neg: Negative. Pos: Positive. Buf: Buffer. PLA-PEG: poly(lactic acid)-poly(ethylene glycol) di-block co-polymer. h: hour. d: days. w: weeks. PLGA: Poly(lactic-co-glycolic) acid. A significant comparison was performed using an ordinary one-way ANOVA followed by Tukey’s multiple comparison tests between M0 and the rest of the groups. p-values < 0.05 were considered statistically significant (*). When no asterisk is displayed, not significative differences were observed. Columns represent the mean ± standard deviation (n = 6).

**Supplementary Table 1. Tg, Tcc, Tm and Xc values of the CLX-releasing prototypes analyzed by DSC and XRD.**

| Sample | Tg (ºC) | Tm (ºC) | Xc (%) |
| --- | --- | --- | --- |
| CLX | - | 161 | - |
| PLLA | 64 | 179 | 5.07 |
| PLLA - CLX | 59 | 174 | 2.85 |
| PCL | -65 to-60* | 61 | 14.14 |
| PCL - CLX | -65 to -60* | 58 | 16.21 |
| PLLA/PCL | 62 | 180 | 14.87 |
| PLLA/PCL - CLX | 58 | 175 | 7.10 |

**Abbreviations:** CLX: Celecoxib. PLLA: poly(L-lactide). PCL: poly(caprolactone). Tg: Glass transition temperature. Tcc: Cold crystallization temperature. Tm: Melting temperature. Xc: Crystallinity.

*Value not observed in Supplementary Figure 4, obtained from [80].

**Supplementary Table 2. Tg, Tcc, Tm and Xc values of the DEX-releasing prototypes analyzed by DSC and XRD.**

| Sample | Tg (ºC) | Tm (ºC) | Xc (%) |
| --- | --- | --- | --- |
| DEX | - | 260 | - |
| PLGA | 44-48 | - | - |
| PLGA – DEX | 44-48 | - | 1.89 |
| PLA-PEG | 48 | - | 6.53 |
| PLA-PEG - DEX | 50 | - | 8.62 |
| LMW-PLGA | 44-48 | - | - |
| LMW-PLGA - DEX | 44-48 | - | 1.8 |

**Abbreviations:** DEX: Dexamethasone. PLGA: Poly(lactic-co-glycolic) acid. PLA-PEG: poly(lactic acid)-poly(ethylene glycol) di-block co-polymer. HMW: Low molecular weight. LMW: Low molecular weight. Tg: Glass transition temperature. Tcc: Cold crystallization temperature. Tm: Melting temperature. Xc: Crystallinity. When “-“ appeared in Tg or Tm, the peak corresponding to the temperature could not be observed. When “-“ appeared in the Xc, the crystallinity could not be measured. It occurred with amorphous polymers.
